# A mathematical model of calcium dynamics: Obesity and mitochondria-associated ER membranes

**DOI:** 10.1371/journal.pcbi.1006661

**Published:** 2019-08-22

**Authors:** Jung Min Han, Vipul Periwal

**Affiliations:** Laboratory of Biological Modeling, National Institute of Diabetes and Digestive and Kidney Diseases, National Institutes of Health, Bethesda, Maryland, United States of America; University of Auckland, UNITED STATES

## Abstract

Multiple cellular organelles tightly orchestrate intracellular calcium (Ca^2+^) dynamics to regulate cellular activities and maintain homeostasis. The interplay between the endoplasmic reticulum (ER), a major store of intracellular Ca^2+^, and mitochondria, an important source of adenosine triphosphate (ATP), has been the subject of much research, as their dysfunction has been linked with metabolic diseases. Interestingly, throughout the cell’s cytosolic domain, these two organelles share common microdomains called mitochondria-associated ER membranes (MAMs), where their membranes are in close apposition. The role of MAMs is critical for intracellular Ca^2+^ dynamics as they provide hubs for direct Ca^2+^ exchange between the organelles. A recent experimental study reported correlation between obesity and MAM formation in mouse liver cells, and obesity-related cellular changes that are closely associated with the regulation of Ca^2+^ dynamics. We constructed a mathematical model to study the effects of MAM Ca^2+^ dynamics on global Ca^2+^ activities. Through a series of model simulations, we investigated cellular mechanisms underlying the altered Ca^2+^ dynamics in the cells under obesity. We predict that, as the dosage of stimulus gradually increases, liver cells from obese mice will reach the state of saturated cytosolic Ca^2+^ concentration at a lower stimulus concentration, compared to cells from healthy mice.

## Author summary

It is well known that intracellular Ca^2+^ oscillations carry encoded signals in their amplitude and frequency to regulate various cellular processes, and accumulating evidence supports the importance of the interplay between the ER and mitochondria in cellular Ca^2+^ homeostasis. Miscommunications between the organelles may be involved in the development of metabolic diseases. Based on a recent experimental study that spotlighted a correlation between obesity and physical interactions of the ER and mitochondria in mouse hepatic cells, we constructed a mathematical model as a tool to probe the effects of the cellular changes linked with obesity on global cellular Ca^2+^ dynamics. Our model successfully reproduced the experimental study that observed a positive correlation between an increase in ER-mitochondrial junctions and the magnitude of mitochondrial Ca^2+^ responses. We postulate that hepatic cells from lean animals exhibit Ca^2+^ oscillations that are more robust under higher concentrations of stimulus, compared to cells from obese animals.

## Introduction

In most multicellular organisms, calcium (Ca^2+^) is a ubiquitous second messenger that controls a vast array of cellular activities spanning from cell birth to apoptosis [1]. The endoplasmic/sarcoplasmic reticulum (ER/SR) and mitochondria have been the center of attention in the study of intracellular Ca^2+^ dynamics, due to their role as internal Ca^2+^ stores. The SR is mostly found in muscle cells, which are not the subject of this paper, so we only refer to the ER. It has been suggested that dysfunction of Ca^2+^ regulation in the ER and/or mitochondria leads to disrupted cellular homeostasis, and is associated with pathological processes, including metabolic diseases and neurodegenerative diseases [2–7].

Upon agonist stimulation, almost all types of cells exhibit fluctuations in cytosolic Ca^2+^ concentration, phenomena often referred to as Ca^2+^ oscillations, with signals encoded in oscillation frequencies and amplitudes. Among many cellular compartments, the ER, whose internal Ca^2+^ concentration is three to four orders of magnitude larger than that of the cytosol in resting condition, is considered as the main contributor to the generation of Ca^2+^ oscillations. The ER has several types of Ca^2+^ channels on the membrane that release Ca^2+^ once activated. The most well-studied Ca^2+^ release channels are inositol trisphosphate receptors (IPRs) and ryanodine receptors (RyRs). As a high cytosolic Ca^2+^ concentration is toxic and often leads to cell death, released Ca^2+^ is quickly pumped back into the ER lumen through sarco/endoplasmic reticulum Ca^2+^ ATPase (SERCA) pumps, which consume energy to sequester Ca^2+^ against its concentration gradient. Some Ca^2+^ released from the ER can be taken up by mitochondria through the mitochondrial Ca^2+^ uniporter (MCU), and then released back to the cytosol via the sodium/calcium exchanger (NCX). Thus, it is generally accepted that mitochondria have the ability to modulate oscillation frequencies and amplitudes, and consequently, affect the progression of cellular activities [4].

Having a spatially extended membrane network, the ER is often positioned in close proximity with other cellular organelles and forms membrane contact sites. Such sites between the ER and mitochondria are called mitochondria-associated ER membranes (MAMs), and it has been suggested that they play a critical role in Ca^2+^ exchange between the organelles [4, 5]. Since mitochondrial Ca^2+^ regulation is closely linked with adenosine triphosphate (ATP) synthesis and reactive oxygen species (ROS) production [8], understanding the mechanisms underlying the ER-mitochondrial Ca^2+^ crosstalk is of great scientific and physiological interest. A major advantage of MAM formation is that due to its minuscule size, even a small Ca^2+^ flux into the domain would be amplified, which is important for the MCUs as they have a low Ca^2+^ affinity, i.e., they require a high concentration of Ca^2+^ in order to activate.

Arruda et al. [9] reported a positive correlation between obesity and the degree of MAM formation. They also found different expression levels of Ca^2+^ channels between liver cells of lean and obese mice. These findings indicate the possibility of obesity-induced changes in Ca^2+^ dynamics in MAMs, and consequently, in the ER as well as mitochondria. Indeed, liver cells from obese animals showed higher baselines of cytosolic Ca^2+^ concentration and mitochondrial Ca^2+^ concentration, compared to cells from lean mice. Furthermore, Ca^2+^ transients generated from ATP stimulation led to higher concentration peaks in obese mouse mitochondria. Interestingly, this observation was not accompanied by higher peaks in cytosolic Ca^2+^ concentration, i.e., cells from obese and lean mice exhibited similar ATP-induced rises in cytosolic Ca^2+^ concentration.

Computational models of experimental data have been a valuable tool for understanding the dynamics of intracellular Ca^2+^. Most models have focused on either the ER Ca^2+^ handling [10–13] or mitochondrial Ca^2+^ dynamics [14–17] and only a handful of them have integrated the dynamics from both organelles [18, 19]. Recently, there have been an increasing number of studies that combined both experimental and theoretical approaches to probe the cellular mechanisms underlying Ca^2+^ crosstalk between the ER and mitochondria. The model proposed by Szopa et al. [20] assumes that due to the minuscule volume of MAMs, the MCUs in MAMs sense Ca^2+^ concentration in the ER. Thus, the MCU Ca^2+^ flux in their model is essentially direct Ca^2+^ flow from the ER. Using numerical methods, they investigated the effects of this flow on the shape (bursting) and period of Ca^2+^ oscillations, and observed that mitochondrial Ca^2+^ concentrations tend to a high level in some regions of parameter space. Another recent model by Qi et al. [21] considers a range of possible distances between the IPRs and MCUs in MAMs, and expresses Ca^2+^ concentration in MAMs as a solution to a linearized reaction-diffusion equation. In this model, the concentration of Ca^2+^ that is sensed by the MCUs in MAMs depends on the distance of the MCUs from the point source (a cluster of IPRs) and how fast Ca^2+^ diffuses in MAMs. The authors showed that Ca^2+^ signals can be significantly modulated by this distance, and determined an optimal distance between the IPRs and MCUs for effective Ca^2+^ exchange for the generation of Ca^2+^ oscillations. On the other hand, Wacquier et al. [22] published a model that associates Ca^2+^ oscillations with mitochondrial metabolism, and investigated the role of mitochondrial Ca^2+^ fluxes on the oscillation frequency. Their model modified one of the parameters that describes the Ca^2+^ concentration for the activation of the MCUs to a lower concentration than the one originally suggested by Magnus and Keizer [16]. By doing so, they implicitly included MAMs, with the following assumption: MCUs are activated at the average concentration of Ca^2+^ in the whole cytosol (including MAMs). They found that mitochondrial Ca^2+^ fluxes can modulate the frequency of Ca^2+^ oscillations.

Here, we construct a mathematical model to investigate the cellular mechanisms underlying the altered mitochondrial Ca^2+^ dynamics observed in obese mice. The model extends the model of Wacquier et al. [22], and explicitly includes Ca^2+^ dynamics in MAMs. We incorporated the model structure proposed by Penny et al. [23], wherein the cytosol is compartmentalized to two separate domains: the bulk cytosol and membrane contact sites between organelles. Rather than expressing the Ca^2+^ concentration in MAMs as an algebraic function of that of the cytosol, we model it as a dynamic variable that is determined by influxes and effluxes of the domain. We investigated how Ca^2+^ signals are affected by the obesity-related changes in Ca^2+^ channel expression levels.

## Materials and models

The full model is a fifteen-dimensional system of ordinary differential equations (ODEs). Using the quasi-steady state approximation, a standard reduction technique for systems with multiple timescales, we reduce the model dimension to eleven. The non-dimensionalization of the full model is explained in S1 Appendix.

A better way of understanding the model structure is to consider the model as two sub-models that are coupled by a common factor, Ca^2+^. One of the sub-models describes intracellular Ca^2+^ dynamics, while the other models mitochondrial metabolic pathways and membrane potential.

### Ca^2+^ dynamics

**Fig 1:**
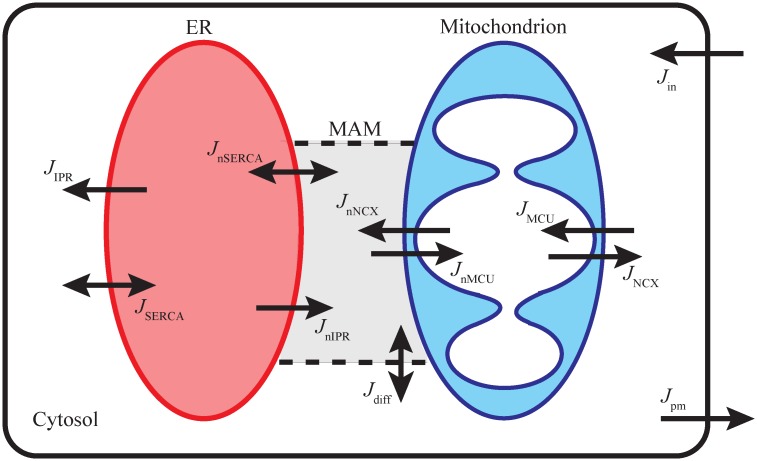
Schematic diagram of Ca^2+^ dynamics described by the model. The ER releases Ca^2+^ to the cytosol and MAM; the fluxes are denoted as *J*_IPR_ and *J*_nIPR_, respectively. The cytosolic Ca^2+^ is pumped back into the ER lumen via SERCA (*J*_SERCA_), and the Ca^2+^ in MAM are also pumped back into the ER (*J*_nSERCA_). Mitochondria uptake Ca^2+^ from the cytosol (*J*_MCU_) and MAM (*J*_nMCU_), and mitochondrial Ca^2+^ is exchanged with Na^+^ in the cytosol (*J*_NCX_) and MAM (*J*_nNCX_). Ca^2+^ can freely diffuse between the cytosol and MAM (*J*_diff_). The total Ca^2+^ concentration is regulated by the plasma membrane influx (*J*_in_) and efflux (*J*_pm_).

We first compartmentalized the cellular domain into four separate regions: the ER, the bulk cytosol, a mitochondrion, and MAM, and assumed that Ca^2+^ concentration within each region, denoted by *C*_*ER*_, *C*_*cyt*_, *C*_*mito*_, and *C*_*MAM*_, respectively, is homogeneous and is determined by Ca^2+^ influxes and effluxes going in and out of that region. Fig 1 shows a schematic diagram of the compartments and Ca^2+^ fluxes in the model. The total intracellular Ca^2+^ concentration, *C*_*t*_, is governed by an interplay of Ca^2+^ fluxes across the plasma membrane. We assumed *C*_*t*_ to be the sum of all compartments’ concentrations. Then, *C*_*ER*_ can be written as:

$$\begin{array}{c} C_{ER}=R_{V2}f_{c}(C_{t}-\frac{1}{R_{V1}f_{c}}C_{MAM}-\frac{1}{f_{c}}C_{cyt}-\frac{1}{R_{V3}f_{m}}C_{mito}), \end{array}$$ (1)

where the *f*’s represent the fraction of free Ca^2+^ that is not bound by buffers. The *R*_v_’s account for compartment volume differences and are defined as:

$$\begin{array}{c} R_{V1}=\frac{\text{total}\;\text{cytosolic}\;\text{volume}}{\text{total}\;\text{MAM}\;\text{volume}},\\ R_{V2}=\frac{\text{total}\;\text{cytosolic}\;\text{volume}}{\text{total}\;\text{ER}\;\text{volume}},\\ R_{V3}=\frac{\text{total}\;\text{cytosolic}\;\text{volume}}{\text{total}\;\text{mitochondrial}\;\text{volume}}. \end{array}$$

Given that the mitochondrial outer membrane is freely permeable to small molecules, such as Ca^2+^, through the voltage dependent anion channel (VDAC), we assume that Ca^2+^ concentration in the inter-membrane space is equivalent to that of the cytosol. Thus, there is one effective layer of impermeable boundary, the mitochondrial inner membrane, that separates cytosolic Ca^2+^ from mitochondrial Ca^2+^.

The Ca^2+^ dynamics part of the model consists of the following ordinary differential equations (ODEs):

$$\begin{array}{l} \frac{d}{dt}C_{cyt}=f_{c}[(1-R_{\text{S1}})(J_{\text{IPR}}-J_{\text{SERCA}})\\ \;+\frac{1-R_{\text{S2}}}{R_{\text{V3}}}(J_{\text{NCX}}-J_{\text{MCU}})+J_{\text{diff}}+J_{\text{in}}-J_{\text{pm}}] \end{array}$$ (2)

$$\begin{array}{cc} & \frac{d}{dt}C_{MAM}=f_{c}[R_{V1}R_{S1}\left(J_{\text{nIPR}}-J_{\text{nSERCA}}\right)\\ & \;+\frac{R_{V1}R_{S2}}{R_{V3}}\left(J_{\text{nNCX}}-J_{\text{nMCU}}\right)-R_{V1}J_{\text{diff}}] \end{array}$$ (3)

$$\begin{array}{cc} & \frac{d}{dt}C_{mito}=f_{m}[R_{S2}\left(J_{\text{nMCU}}-J_{\text{nNCX}}\right)+\left(1-R_{S2}\right)\left(J_{\text{MCU}}-J_{\text{NCX}}\right)] \end{array}$$ (4)

$$\begin{array}{cc} & \frac{d}{dt}C_{t}=J_{\text{in}}-J_{\text{pm}} \end{array}$$ (5)

$$\begin{array}{cc} & \frac{d}{dt}P=\tau_{p}\left(P_{s}-P\right)+\text{pulse} \end{array}$$ (6)

$$\begin{array}{cc} & \frac{d}{dt}h_{42}=\lambda_{h_{42}}\left(h_{42}^{\infty }-h_{42}\right) \end{array}$$ (7)

$$\begin{array}{cc} & \frac{d}{dt}h_{n42}=\lambda_{h_{n42}}\left(h_{n42}^{\infty }-h_{n42}\right) \end{array}$$ (8)

*P* represents the homogeneous concentration of IP_3_ in the bulk cytosol and the MAM. *h*_42_ and *h*_n42_ denote the activation variables of the IPRs in the bulk cytosol and the MAM, respectively. The *R*_S_’s are surface ratios, defined as

$$R_{S1}=\frac{\text{surface}\;\text{area}\;\text{of}\;\text{the}\;\text{ER}\;\text{that}\;\text{adjoins}\;\text{the}\;\text{MAM}}{\text{the}\;\text{total}\;\text{ER}\;\text{surface}\;\text{area}},$$ (9)

$$R_{S2}=\frac{\text{surface}\;\text{area}\;\text{of}\;\text{mitochondrion}\;\text{that}\;\text{adjoins}\;\text{the}\;\text{MAM}}{\text{the}\;\text{total}\;\text{mitochondrion}\;\text{surface}\;\text{area}};$$ (10)

see Table 1 for their values. Short descriptions for the J_*_ Ca^2+^ fluxes are given below.

- *J*_IPR_ and *J*_nIPR_: IPR Ca^2+^ flux into the bulk cytosol and MAMs from the ER
- *J*_SERCA_ and *J*_nSERCA_: SERCA pump Ca^2+^ flux into the ER from the bulk cytosol and MAMs
- *J*_NCX_ and *J*_nNCX_: NCX Ca^2+^ flux from mitochondria into the bulk cytosol and MAMs
- *J*_MCU_ and *J*_nMCU_: MCU Ca^2+^ flux into mitochondria from the bulk cytosol and MAMs
- *J*_diff_: Ca^2+^ diffusion between the bulk cytosol and MAMs
- *J*_in_: Ca^2+^ influx across the plasma membrane through store-operated Ca^2+^ channels (SOCCs), receptor-operated Ca^2+^ channels (ROCCs), and a constant small influx
- *J*_pm_: Ca^2+^ efflux across the plasma membrane through the plasma membrane Ca^2+^ ATPase (PMCA) pumps

**Table 1:** Parameter values of the model. The parameters with ★ in the reference column are chosen to reproduce some of experimental data reported by Arruda et al. [9]. The parameters with † in the reference column are modified from the original values proposed by Wacquier et al. [22]. The original values are shown at the bottom of the table.

Parameter	Value (Unit)	Description	Ref.
Parameters for the Ca^2+^ and IP_3_ concentration equations			
R_V1_	2000	the volume ratio between the cytosol and MAM	★
R_V2_	10	the volume ratio between the cytosol and the ER	[22]
R_V3_	15	the volume ratio between the cytosol and mitochondria	†
R_S1_	0.15	the proportion of the ER membrane surface that adjoins the MAM	[9]
R_S2_	0.15	the proportion of mitochondrial membrane surface that adjoins the MAM	[29]
*ω*_*c*_	0.001 (s^−1^)	intracellular Ca^2+^ diffusion rate	★
*τ*_*p*_	0.1 (s^−1^)	the rate at which IP_3_ concentration reaches its equilibrium	★
*f*_*c*_	0.01	fraction of free Ca^2+^ in the cytosol, MAM, and ER lumen that are not buffered	[18]
*f*_*m*_	0.0003	fraction of free Ca^2+^ in mitochondria that are not buffered	[18]
*k*_IPR_	0.3 (s^−1^)	coefficient of the IPR flux entering the bulk cytosol and the MAM	★
*k*_nIPR_	0.15 (s^−1^)	coefficient of the IPR flux entering the MAM	★
*V*_SERCA_	30 (*μ*M s^−1^)	the maximum SERCA flux from the bulk cytosol	★
*V*_nSERCA_	10 (*μ*M s^−1^)	the maximum SERCA flux from the MAM	★
*K*_SERCA_	0.35 (*μ*M)	half-maximal activating cytosolic Ca^2+^ concentration of SERCA	[30]
$\bar{k}$	1 × 10^−8^	concentrating power of the ATPase	★
*V*_MCU_	0.00001 (*μ*M s^−1^)	coefficient of the MCU flux from the bulk cytosol and the MAM	†
*V*_NCX_	0.5 (*μ*M s^−1^)	coefficient of the NCX flux entering the bulk cytosol and the MAM	†
*K*_1_	19 (*μ*M)	dissociation constant for Ca^2+^ translocation by the MCU	[16]
*K*_2_	0.38 (*μ*M)	dissociation constant for MCU activation by Ca^2+^	[16]
*p*_1_	0.1 (mV^−1^)	coefficient of MCU activity dependence on voltage	[22]
*p*_2_	0.016 (mV^−1^)	coefficient of NCX activity dependence on voltage	[22]
*V*_SOCC_	0.8 (*μ*M s^−1^)	the maximum SOCC flux entering the cytosol	★
*K*_SOCC_	100 (*μ*M)	half-maximal inhibiting ER Ca^2+^ concentration of SOCC	★
*V*_ROCC_	0.25 (s^−1^)	coefficient of the ROCC entering the cytosol	★
*k*_leakin_	0.0019 (*μ*M s^−1^)	plasma membrane leak influx	★
*V*_pm_	0.2 (*μ*M s^−1^)	the maximum PMCA efflux exiting the cytosol	★
*K*_pm_	0.45 (*μ*M)	half-maximal activating cytosolic Ca^2+^ concentration of PMCA	[31]
Parameters for the IPR model equations			
*q*_26_	10500 (s^−1^)	transition rate from *C*_2_ to *O*_6_	[24]
*q*_62_	4010 (s^−1^)	transition rate from *O*_6_ to *C*_2_	[24]
*L*_IPR_	0.02 (s^−1^)	the rate at which *h*_42_ and *h*_n42_ reach their equilibria, if the channel is in the park mode	★
*H*_IPR_	0.1 (s^−1^)	the rate at which *h*_42_ and *h*_n42_ reach their equilibria, if the channel is in the drive mode	★
*C*_*p*0_	700 (*μ*M)	Ca^2+^ concentration at the pore of an open IPR	★
Parameters for the mitochondrial metabolic pathway equations			
$N_{mito}^{\text{tot}}$	250 (*μ*M)	the total mitochondrial pyridine nucleotide concentration	[22]
$A_{mito}^{\text{tot}}$	15000 (*μ*M)	the total mitochondrial adenine nucleotide concentration	[22]
$A_{cyt}^{\text{tot}}$	2500 (*μ*M)	the total cytosolic adenine nucleotide concentration	[22]
*C*_*p*_	1.8 (*μ*M mV^−1^)		[22]
*L*_MCU_	50	allosteric equilibrium constant for uniporter conformations	[32]
*a*_1_	20	scaling factor between NADH oxidation and change in mitochondrial membrane voltage	[22]
*a*_2_	3.43	scaling factor between ADP phosphorylation and change in mitochondrial membrane voltage	[15]
*V*_hyd_	150 (*μ*M s^−1^)	maximum rate of ATP hydrolysis	†
*K*_hyd_	1000 (*μ*M)	half-maximal activating cytosolic ATP concentration of ATP hydrolysis	[22]
*V*_ant_	5000 (*μ*M s^−1^)	rate coefficient of the adenine nucleotide translocator	[15]
*α*_*c*_	0.111	cytosolic ADP and ATP buffering coefficient	[18]
*α*_*m*_	0.139	mitochondrial ADP and ATP buffering coefficient	[18]
*F*	96480 (C mol^−1^)	Faraday constant	
*R*	8315 (mJ mol^−1^ K^−1^)	perfect gas constant	
*T*	310.16 (K)	temperature	
*V*_F1FO_	35000 (*μ*M s^−1^)	rate coefficient of the F_1_F_O_-ATPase	[15]
*k*_gly_	450 (*μ*M s^−1^)	rate coefficient of glycolysis	[15]
*k*_o_	600 (*μ*M s^−1^)	rate coefficient of NADH oxidation by ETC	[15]
*V*_agc_	100 (*μ*M s^−1^)	rate coefficient of NADH production	†
*K*_agc_	0.14 (*μ*M)	dissociation constant of cytosolic Ca^2+^ from AGC	[22]
*p*_4_	0.005 (mV^−1^)	coefficient of AGC activity dependence on voltage	†
*q*_1_	1	Michaelis-Menten-like constant for NAD^+^ consumption by the TCA	[15]
*q*_2_	0.1 (*μ*M)	half-maximal activating mitochondrial Ca^2+^ concentration of the TCA	[22]
*q*_3_	100 (mV)	Michaelis-Menten constant for NADH consumption by the ETC	[15]
*q*_4_	177 (mV)	coefficient of ETC activity dependence on voltage	[15]
*q*_5_	5 (mV)	coefficient of ETC activity dependence on voltage	[15]
*q*_6_	10000 (*μ*M)	inhibition constant of ATPase activity by ATP	[15]
*q*_7_	190 (mV)	coefficient of ATPase activity dependence on voltage	[15]
*q*_8_	8.5 (mV)	coefficient of ATPase activity dependence on voltage	[15]
*q*_9_	2 (*μ*M s^−1^ mV^−1^)	proton leak dependence on voltage	[15]
*q*_10_	−30 (*μ*M s^−1^)	rate coefficient of voltage-independent proton leak	[15]

^†^: *R*_V3_ = 1/0.0733, *V*_MCU_ = 0.0006 *μ*M s^−1^, *V*_NCX_ = 0.35 *μ*M s^−1^, *V*_hyd_ = 100 *μ*M s^−1^, *V*_agc_ = 25 *μ*M s^−1^, *p*_4_ = 0.01 mV^−1^.

#### IPR model

We incorporated the IPR model proposed in Cao et al. [24], which assumes that the receptors are either in drive mode when they are mostly open, or in park mode when they are mostly closed. The drive mode has one open state (*O*_6_) and one closed state (*C*_2_), while there is one closed state (*C*_4_) in the park mode. The transition rates between the modes are denoted by *q*_24_ (drive → park) and *q*_42_ (park → drive), and the rates between the states within the drive mode are *q*_26_ and *q*_62_. The open probability of the drive mode is *q*_26_/(*q*_26_ + *q*_62_) (≈ 70%). While the IPRs in MAMs share the rate *q*_24_ with those in the cytosol, they have their own park to drive transition rate, denoted by *q*_*n*42_. *q*_24_, *q*_42_, and *q*_*n*42_ are given by

$$q_{24}=a_{24}+V_{24}\left(1-m_{24}h_{24}\right),$$ (11)

$$q_{42}=a_{42}+V_{42}m_{42}h_{42},$$ (12)

$$q_{n42}=a_{42}+V_{42}m_{n42}h_{n42}.$$ (13)

The *m*’s and *h*’s are gating variables that govern the opening and closing kinetics of the receptors, with the following quasi-equilibria:

$$m_{24}^{\infty }=\frac{C_{p}^{3}}{C_{p}^{3}+k_{24}^{3}},$$ (14)

$$h_{24}^{\infty }=\frac{k_{-24}^{2}}{C_{p}^{2}+k_{-24}^{2}},$$ (15)

$$m_{42}^{\infty }=\frac{C_{cyt}^{3}}{C_{cyt}^{3}+k_{42}^{3}},$$ (16)

$$m_{n42}^{\infty }=\frac{C_{MAM}^{3}}{C_{MAM}^{3}+k_{42}^{3}},$$ (17)

$$h_{42}^{\infty }=\frac{k_{-42}^{3}}{C_{cyt}^{3}+k_{-42}^{3}}$$ (18)

$$h_{n42}^{\infty }=\frac{k_{-42}^{3}}{C_{MAM}^{3}+k_{-42}^{3}}.$$ (19)

*C*_*p*_ = *C*_*p*0_(*C*_*ER*_/680) denotes the concentration of Ca^2+^ at the pore of a receptor. The non-dimensionalization of the full model is given in the S1 Appendix. It shows that *m*_42_, *m*_*n*42_, *m*_24_, and *h*_24_ evolve on a faster timescale than the other variables. We applied the quasi-steady-state reduction technique and assumed that these gating variables reach their quasi-equilibria instantaneously. The rates at which *h*_42_ and *h*_n42_ approach their equilibria, $\lambda_{h_{42}}$ and $\lambda_{h_{n42}}$, respectively, describe average rates between the rate at which the receptors in the drive mode are inhibited by a high Ca^2+^ concentration, denoted by *H*_*IPR*_, and the slow recovery rate from the inhibition for those in the park mode, denoted by *L*_*IPR*_. Hence,

$$\lambda_{h_{42}}=\left(1-D\right)L_{\text{IPR}}+DH_{\text{IPR}}$$ (20)

$$\lambda_{h_{n42}}=\left(1-D_{n}\right)L_{\text{IPR}}+D_{n}H_{\text{IPR}}.$$ (21)

The following expressions for the *V*’s, *a*’s, and *k*’s were chosen by Cao et al. [24] to reproduce the means of *q*_24_ and *q*_42_ distributions reported by Siekmann et al. [25].

$$\begin{array}{cc} V_{24}=62+880/(P^{2}+4) & \;a_{24}=1+5/(P^{2}+0.25)\\ k_{24}=0.35 & k_{-24}=80\\ V_{42}=110P^{2}/\left(P^{2}+0.01\right) & \;a_{42}=1.8P^{2}/\left(P^{2}+0.34\right)\\ k_{42}=0.49+0.543P^{3}/\left(P^{3}+64\right) & k_{-42}=0.41+25P^{3}/\left(P^{3}+274.6\right) \end{array}$$ (22)

The open probability of the IPRs in the cytosol, *O*_IPR_, and that in the MAM, *O*_nIPR_, are defined as

$$O_{\text{IPR}}=\frac{q_{26}}{q_{62}+q_{26}}D,$$ (23)

$$O_{\text{nIPR}}=\frac{q_{26}}{q_{62}+q_{26}}D_{n}$$ (24)

where *D* and *D*_n_,

$$\begin{array}{ccc} D & = & \frac{q_{42}\left(q_{62}+q_{26}\right)}{q_{42}q_{62}+q_{42}q_{26}+q_{24}q_{62}}, \end{array}$$ (25)

$$\begin{array}{ccc} D_{n} & = & \frac{q_{n42}\left(q_{62}+q_{26}\right)}{q_{n42}q_{62}+q_{n42}q_{26}+q_{24}q_{62}}, \end{array}$$ (26)

represent the proportions of the IPRs in the cytosol and MAMs that are in the drive mode, respectively. Then the IPR fluxes are modeled as:

$$J_{\text{IPR}}=k_{\text{IPR}}O_{\text{IPR}}\left(C_{ER}-C_{cyt}\right),$$ (27)

$$J_{\text{nIPR}}=k_{\text{nIPR}}O_{\text{nIPR}}\left(C_{ER}-C_{MAM}\right).$$ (28)

*k*_IPR_ and *k*_nIPR_ represent the activity levels of the receptors in the bulk ER and MAMs, respectively. Arruda et al. [9] reported that, in wild type mouse hepatocytes, the expression level of IPRs is higher in the bulk ER than that in MAMs. Based on this, we assumed the following:

$$\begin{array}{c} k_{\text{nIPR}}=0.5k_{\text{IPR}}. \end{array}$$ (29)

We note that hepatocytes from obese mice may have a different IPR expression profile.

The diffusion flux between the MAM and the cytosol is a linear function of the concentration difference,

$$\begin{array}{c} J_{\text{diff}}=\omega_{c}\left(C_{MAM}-C_{cyt}\right). \end{array}$$ (30)

Upon the stimulation of ER Ca^2+^ release, Ca^2+^ concentration in MAMs is expected to be much higher than that of the bulk cytosol, due to the volume difference between the compartments. Thus, Ca^2+^ diffusion between the compartments, if any, should be from MAMs to the bulk cytosol. However, most of the Ca^2+^ released to MAMs is expected to be quickly pumped back into the ER by SERCA or transported to mitochondria through MCUs, before it can reach the furthest domain in the bulk cytosol. We assumed that the effect of Ca^2+^ diffusion from MAMs to the bulk cytosol is insignificant to the Ca^2+^ dynamics, and set *ω*_*c*_ ≪ 1.

As the cytosolic Ca^2+^ concentration increases, SERCA pumps are activated to sequester Ca^2+^ back into the ER. The SERCA fluxes include a reverse reaction to account for the net pump flux approaching zero when the ER lumen Ca^2+^ concentration is high enough. The fluxes are given by

$$J_{\text{SERCA}}=V_{\text{SERCA}}\frac{C_{cyt}^{2}-\bar{k}C_{ER}^{2}}{K_{\text{SERCA}}^{2}+C_{cyt}^{2}},$$ (31)

$$J_{\text{nSERCA}}=V_{\text{nSERCA}}\frac{C_{MAM}^{2}-\bar{k}C_{ER}^{2}}{K_{\text{SERCA}}^{2}+C_{MAM}^{2}}.$$ (32)

We set $\bar{k}⪡1$ to satisfy *C*_*cyt*_ ≪ *C*_*ER*_ at steady state.

The cell plasma membrane influx (*J*_in_) and efflux (*J*_pm_) control the total intracellular Ca^2+^ concentration. We follow [26] to model these fluxes. *J*_in_ consists of three fluxes:

$$\begin{array}{c} J_{\text{in}}=J_{\text{leakin}}+J_{\text{SOCC}}+J_{\text{ROCC}}, \end{array}$$ (33)

where

$$J_{\text{SOCC}}=V_{\text{SOCC}}\frac{K_{\text{SOCC}}^{4}}{K_{\text{SOCC}}^{4}+C_{\text{ER}}^{4}},$$ (34)

$$J_{\text{ROCC}}=V_{\text{ROCC}}\cdot P,$$ (35)

and *J*_leakin_ is a small contant. *J*_pm_ is modeled as

$$\begin{array}{c} J_{\text{pm}}=V_{\text{pm}}\frac{C_{cyt}^{2}}{C_{cyt}^{2}+K_{\text{pm}}^{2}}. \end{array}$$ (36)

We follow Wacquier et al. [22] in modeling the MCU and the NCX fluxes,

$$J_{\text{MCU}}=V_{\text{MCU}}\frac{\frac{C_{cyt}}{K_{1}}(1+\frac{C_{cyt}}{K_{1}}{)}^{3}e^{p_{1}V_{m}}}{(1+\frac{C_{cyt}}{K_{1}}{)}^{4}+\frac{L_{\text{MCU}}}{{\left(1+\frac{C_{cyt}}{K_{2}}\right)}^{2.8}}},$$ (37)

$$J_{\text{nMCU}}=V_{\text{MCU}}\frac{\frac{C_{MAM}}{K_{1}}(1+\frac{C_{MAM}}{K_{1}}{)}^{3}e^{p_{1}V_{m}}}{(1+\frac{C_{MAM}}{K_{1}}{)}^{4}+\frac{L_{\text{MCU}}}{{\left(1+\frac{C_{MAM}}{K_{2}}\right)}^{2.8}}},$$ (38)

$$J_{\text{NCX}}=V_{\text{NCX}}(\frac{C_{mito}}{C_{cyt}})e^{p_{2}V_{m}},$$ (39)

$$J_{\text{nNCX}}=V_{\text{NCX}}(\frac{C_{mito}}{C_{MAM}})e^{p_{2}V_{m}}.$$ (40)

The detailed modeling of the mitochondrial membrane potential, denoted by *V*_*m*_, is explained in the following section, along with other mitochondrial metabolic pathway variables. There is no concrete experimental evidence that favors either a particular distribution of MCUs and NCXs or qualitatively different channel activities on the MAM sector of the mitochondrial membrane, compared to the membrane facing the cytosol. Thus, we assumed that the rate constants of MCUs and NCXs are the same across the mitochondrial membrane.

Notably, Wacquier et al. [22] set the parameter *K*_1_ to 6 *μ*M, but this represents the average level of Ca^2+^ in the whole cytosol, including MAMs, when the concentration reaches a physiologically reasonable level in MAMs. Since we considered MAMs explicitly in the model, we set *K*_1_ to 19 *μ*M, as originally proposed by Magnus et al. [16].

### Mitochondrial metabolic pathway model

The main function of mitochondria is to create ATP by oxidative phosphorylation. Due to this particular role, mitochondria are the powerhouse of the cell. The mitochondrial metabolic pathway is initiated by the uptake of pyruvate, which is the end product of cytosolic glycolysis. Pyruvate in the mitochondrial matrix then enters the tricarboxylic acid (TCA) cycle, also known as the citric acid cycle or the Krebs cycle, to generate the reducing agent NADH that has electrons with a high transfer potential. The concentration of mitochondrial NADH can also be increased by the activity of the malate-aspartate shuttle (MAS). NADH then goes through the electron transport chain (ETC), where the electrons are separated and used to drive protons (H^+^) across the inner membrane and generate a proton gradient between the intermembrane space and mitochondrial matrix. As protons accumulate in the intermembrane space, the potential gradient across the inner membrane is used by the F1FO-ATPase to convert mitochondrial adenosine diphosphate (ADP) to ATP via phosphorylation. The produced ATP is then transported to the cytosol by adenine nucleotide translocases (ANT), which carry out the exchange of cytosolic ADP and mitochondrial ATP across the inner mitochondrial membrane.

Ca^2+^ is an important component in mitochondrial metabolism, as it promotes the production of NADH. An increase in mitochondrial Ca^2+^ concentration upregulates the TCA cycle, and an increase in cytosolic Ca^2+^ concentration stimulates the aspartate-glutamate carrier (AGC), a protein involved in the MAS. We combined the calcium model with a model of mitochondrial metabolic pathways proposed by Wacquier et al. [22]:

$$\frac{d}{dt}ADP_{c}=I_{\text{hyd}}-\frac{I_{\text{ant}}}{R_{V3}}$$ (41)

$$\frac{d}{dt}ADP_{m}=I_{\text{ant}}-I_{F1\text{FO}}$$ (42)

$$\frac{d}{dt}N=I_{\text{pdh}}-I_{o}+I_{\text{agc}}$$ (43)

$$\begin{array}{c} \begin{array}{cc} \frac{d}{dt}V_{m} & =\frac{1}{C_{p}}(a_{1}I_{o}-a_{2}I_{F1\text{FO}}-I_{\text{ant}}-I_{\text{Hleak}}\\ & \;-\left(1-R_{S2}\right)\left(J_{\text{NCX}}+2J_{\text{MCU}}\right)-R_{S2}\left(J_{\text{nNCX}}+2J_{\text{nMCU}}\right)-I_{\text{agc}}) \end{array} \end{array}$$ (44)

The variables *ADP*_*c*_ and *ADP*_*m*_ measure ADP concentrations in the cytosol and mitochondrion, while *N* is the concentration of mitochondrial NADH. *V*_*m*_ models the voltage difference across the inner mitochondrial membrane. The I_*_ rates are:

- *I*_hyd_: rate of ATP hydrolysis
- *I*_ant_: rate of the ADP/ATP translocator
- *I*_F1FO_: rate of ADP phosphorylation
- *I*_pdh_: the production rate of NADH by the pyruvate dehydrogenase
- *I*_o_: rate of NADH oxidation
- *I*_agc_: the production rate of NADH from the MAS
- *I*_Hleak_: the ohmic mitochondrial proton leak

The model suggests the conservation of the following ion concentrations: total NADH (oxidized and reduced), mitochondrial di- and triphosphorylated adenine nucleotides, and cytosolic di- and triphosphorylated adenine nucleotides. Mathematically speaking,

$$N_{mito}^{\text{tot}}=N+NAD,$$ (45)

$$A_{mito}^{\text{tot}}=ADP_{m}+ATP_{m},$$ (46)

$$A_{cyt}^{\text{tot}}=ADP_{c}+ATP_{c}.$$ (47)

Other functions of the mitochondrial model in Wacquier et al. [22] are reproduced below for convenience.

$$I_{\text{hyd}}=\frac{\left(1-R_{S1}\right)J_{\text{SERCA}}+R_{S1}J_{\text{nSERCA}}}{2}+V_{\text{hyd}}\frac{ATP_{c}}{ATP_{c}+K_{\text{hyd}}}$$ (48)

$$I_{\text{ant}}=V_{\text{ant}}\frac{1-\frac{\alpha_{c}ATP_{c}ADP_{m}}{\alpha_{m}ADP_{c}ATP_{m}}e^{\frac{FV_{m}}{RT}}}{\left(1+\alpha_{c}\frac{ATP_{c}}{ADP_{m}}e^{-0.5\frac{FV_{m}}{RT}})(1+\frac{ADP_{m}}{\alpha_{m}ATP_{m}}\right)}$$ (49)

$$I_{F1\text{FO}}=V_{F1\text{FO}}(\frac{q_{6}}{q_{6}+ATP_{m}})(1+e^{\frac{q_{7}-V_{m}}{q_{8}}}{)}^{-1}$$ (50)

$$I_{\text{pdh}}=k_{\text{gly}}\frac{1}{q_{1}+\frac{N}{NAD}}\frac{C_{mito}}{q_{2}+C_{mito}}$$ (51)

$$I_{o}=k_{o}\frac{N}{q_{3}+N}(1+e^{\frac{V_{m}-q_{4}}{q_{5}}}{)}^{-1}$$ (52)

$$I_{\text{agc}}=V_{\text{agc}}\frac{C_{cyt}}{K_{\text{agc}}+C_{cyt}}\frac{q_{2}}{q_{2}+C_{mito}}e^{p_{4}V_{m}}$$ (53)

$$I_{\text{Hleak}}=q_{9}V_{m}+q_{10}$$ (54)

The model parameters are in Table 1. For modeling purposes, some of the parameters are modified from their original values as in Wacquier et al. [22]. We find these modifications justifiable, as the original values were chosen by the authors to reproduce their experimental data, and hence were not based on any direct physiological evidence.

#### IP_3_ metabolism

A number of studies suggest Ca^2+^ oscillations in hepatocytes can occur at a constant level of IP_3_. In particular, an experimental study reported oscillating Ca^2+^ concentrations in the ER lumen in permeabilized hepatocytes, while the concentration of IP_3_ was clamped at a submaximal concentration [27]. Thus, we assumed that Ca^2+^ oscillations are primarily generated by Ca^2+^ feedback on the opening and closing kinetics of the IPR. For simplicity, we did not consider cellular formation or breakdown of IP_3_ that involves Ca^2+^ or protein kinase C (PKC). Instead, we follow Sneyd et al. [28] and model IP_3_ dynamics as a gradual increase from 0 *μ*M to its steady-state concentration, *P*_s_, at a rate of *τ*_*p*_. However, if hepatocytes were to exhibit IP_3_ oscillations, it is possible to include positive and/or negative Ca^2+^ feedback on IP_3_ metabolism in the model to introduce IP_3_ oscillations as passive reflections of Ca^2+^ oscillations.

Apart from continuous stimulation, the model can be perturbed with a single stimulation in a pulsatile manner. In such a case, cells would be exposed to a certain amount of agonist for a short period of time. While continuous stimulation saturates the concentration of IP_3_ to its steady state, a pulse stimulation produces a sudden increase in IP_3_ concentration, followed by a natural decay at a rate of degradation. In the model, a pulse of stimulation is described by

$$\begin{array}{c} \text{pulse}=MH\left(t-t_{0}\right)H\left(t_{0}+△-t\right), \end{array}$$ (55)

where *H* is the Heaviside function

$$\begin{array}{c} H\left(t-t_{0}\right)=\{\begin{array}{cc} 0 & \text{if}\;t\leq t_{0},\\ 1 & \text{if}\;t>t_{0}, \end{array} \end{array}$$

*M* is the pulse magnitude, *t*_0_ is the time at which the pulse is given, and △ is the pulse duration.

### Numerical simulations

All the numerical simulations presented in this paper were computed with XPPAUT [33].

## Results

This section consists of two parts: model verification and model prediction. In the first part, model behaviors are compared with the experimental data presented in Arruda et al. [9], to show the validity of the model. In the second part, the model is used to simulate Ca^2+^ oscillations in hepatocytes from wild type and genetically obese (*ob*/*ob*) mice, to predict the effects of obesity-related cellular changes on Ca^2+^ dynamics.

### Model verification

#### Up-regulation of MAMs and mitochondrial Ca^2+^ activities

Due to the morphology of MAMs, where the ER and mitochondria are in close contact, it is generally accepted that there is direct Ca^2+^ exchange between the organelles at such sites. Based on this notion, it is reasonable to expect that an increase in the degree of MAM formation up-regulates mitochondrial Ca^2+^ intake via MAMs, and consequently induces larger amplitudes of mitochondrial Ca^2+^ signals. In fact, Arruda et al. [9] used synthetic linkers to mechanically induced more MAMs in hepatocyte-derived mouse Hepa1-6 cells, and observed about 20% higher ATP-stimulated mitochondrial Ca^2+^ peaks, compared to the cells without the linkers. This experiment revealed a correlation between mitochondrial Ca^2+^ activity and ER–mitochondrial interactions.

**Fig 2:**
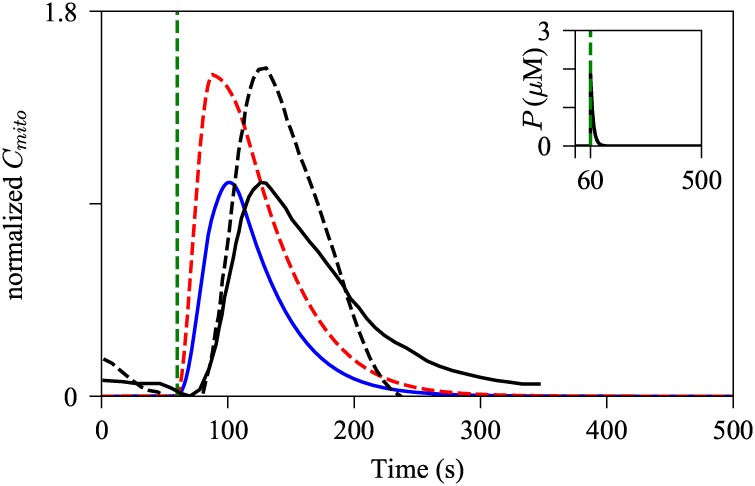
Effects of increased MAMs on amplitude of mitochondrial Ca^2+^ activities. The model was simulated with a pulse of IP_3_, Eq 55 with *M* = 10, *t*_0_ = 60, and Δ = 0.2, shown by the inset graph. The blue solid trajectory was simulated with (*R*_S1_, *R*_S2_) = (0.15, 0.15), while the red dashed spike was generated with the increased MAM surface ratios, (*R*_S1_, *R*_S2_) = (0.48, 0.48). The black solid curve represents experimentally traced mitochondrial Ca^2+^ dynamics in control Hepa 1-6 cells, while the black dashed line describes that in the cells expressed with synthetic linkers. The green dashed line indicates the onset of the pulse in the model, and the time at which the cells were treated with 100 *μ*M of ATP in the experiment. The experimental traces were obtained from Arruda et al. [9].

To determine whether the model can reproduce the experimental observations of Arruda et al. [9], we performed model simulations using two different sets of values for the *R*_S_ parameters, the portions of the ER and mitochondrial membranes that face each other. Rizzuto et al. [29] estimated that about 5–20% of mitochondrial membrane faces MAMs, and based on this we set *R*_S2_ to 0.15. Moreover, Arruda et al. [9] quantified the normalized ER length adjacent to mitochondria to be about 20%. Although the length scale does not directly convert to the surface area scale, it is the qualitative change of the MAM structure that should be reflected in the model simulations. For simplicity, we set *R*_S1_ to be equal to *R*_S2_. The model with (*R*_S1_, *R*_S2_) = (0.15, 0.15) was stimulated with a pulse of IP_3_, generated from Eq 55 with *M* = 10, *t*_0_ = 60, and △ = 0.2. Then the same stimulation was applied to the model with (*R*_S1_, *R*_S2_) = (0.48, 0.48), which mimics the up-regulation of MAMs induced by the synthetic linkers. The values were particularly chosen to reproduce the mitochondrial Ca^2+^ transient peak difference observed in the experiment data published in Arruda et al. [9]. Fig 2 shows the normalized representative experimental data and the corresponding model simulation, plotted on the same axes. The original experimental data were normalized as:

$$\begin{array}{c} C_{mito,normalized}=\frac{C_{mito}-\text{min}\left(C_{mito}^{control}\right)}{\text{max}\left(C_{mito}^{control}\right)-\text{min}\left(C_{mito}^{control}\right)}, \end{array}$$ (56)

where the max($C_{mito}^{control}$) and min($C_{mito}^{control}$) are the maximum and the minimum of the control mitochondrial Ca^2+^ transient fluorescence resonance energy transfer (FRET) ratio traced from control Hepa 1-6 cells. By doing so, the amplitude of the control mitochondrial Ca^2+^ transient was normalized to [0, 1], and the mitochondrial Ca^2+^ transient from the cells expressed with synthetic linkers was normalized with respect to the control transient. For better comparison, we normalized the model outputs in the same notion, using the mitochondrial Ca^2+^ dynamics from the control model as a reference. As shown in Fig 2, the model with the modified parameters generated a higher peak of *C*_*mito*_ transient, and the result is consistent with the experimental observation reported by Arruda et al. Given that the MCU has a low Ca^2+^ affinity, i.e., it requires high concentrations of Ca^2+^ for channel activation, increasing the mitochondrial surface portion that faces MAMs, and thus exposing more MCUs to higher concentrations of Ca^2+^, would lead to augmented MCU Ca^2+^ flux, and consequently, induce larger amplitudes of mitochondrial Ca^2+^ activities.

The Ca^2+^ transients in the model show a faster reaction time to the perturbation, compared to the experimental data. There are several factors that may play a role in this discrepancy. For simplicity, we modeled the stimulation as a pulse of IP_3_, with a fast increase in the concentration of IP_3_ followed by a relatively slow decay. However, the time course of IP_3_ concentration was not measured in the experiment, and thus we cannot verify whether the model’s IP_3_ trajectory mimics the IP_3_ dynamics in the experiment. Furthermore, the model assumes spatially homogeneous mitochondrial Ca^2+^ concentration, whereas in reality, the Ca^2+^ concentration first rises near domains with a dense population of MCUs, then subsequently spreads to other parts of the domain. Although the model accurately reproduced the mitochondrial Ca^2+^ peak increase associated with the upregulation of MAMs, it certainly lacks finesse for simulating the precise timescale of the mitochondrial Ca^2+^ spikes.

#### Dynamics of the mitochondrial variables

A number of studies traced cytosolic Ca^2+^ concentration and mitochondrial metabolic products, such as ATP and NADH, to investigate the coordination of calcium signals and the target responses. Recorded by Gaspers et al. [34], the dynamics of mitochondrial NADH was shown to be closely coupled to that of cytosolic Ca^2+^. The study reported a fast single cytosolic Ca^2+^ spike accompanied by an increase in the mitochondrial NADH concentration and then a prolonged phase of NADH oxidation. Moreover, continuous Ca^2+^ oscillations were paired with sustained mitochondrial NADH metabolic cycles at a raised basal concentration. In follow-up studies, Gaspers et al. [35, 36] observed simultaneous increases in cytosolic Ca^2+^ concentration and mitochondrial membrane potential, which was the opposite of what the authors had expected. In a separate study, histamine-dependent mitochondrial Ca^2+^ signals were accompanied by relatively slower ATP responses in both cytosol and mitochondria [37].

**Fig 3:**
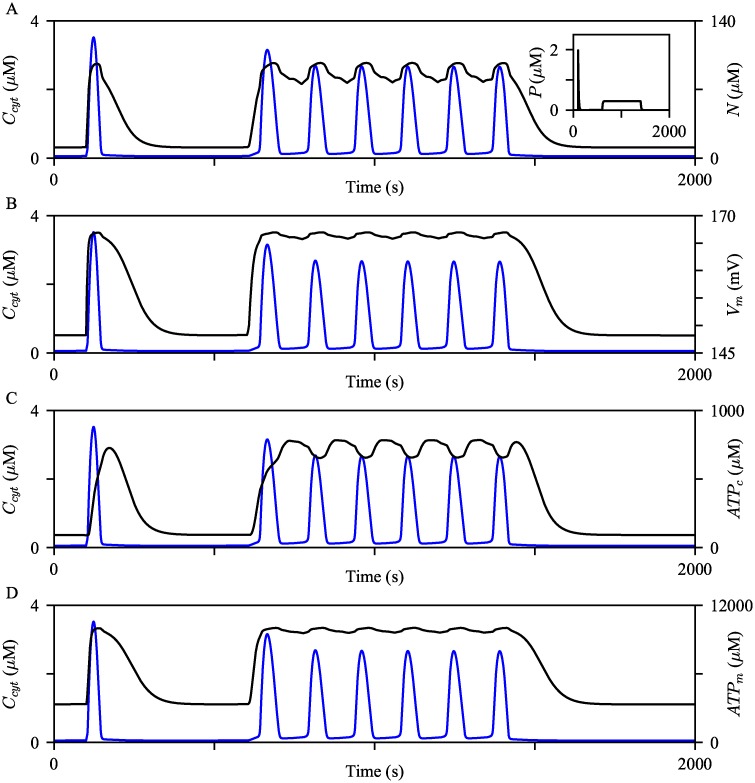
Evolution of mitochondrial variables. The model was simulated with two different styles of IP_3_ dynamics, shown by the inset graph of (A). The resulting behaviors of *C*_*cyt*_ are shown by the blue curves. The black curves show (A) *V*_*m*_, (B) *N*, (C) *ATP*_*c*_, and (D) *ATP*_*m*_ timeseries concurrent with the *C*_*cyt*_ timeseries.

We examined behaviors of the mitochondrial variables of the model to confirm that the model can of reproduce the experimental observations described above. The model was first simulated with a pulse of IP_3_, Eq 55 with *M* = 5, *t*_0_ = 100, and Δ = 0.2. Then the second phase of IP_3_ dynamics was generated from the following condition:

$$\begin{array}{c} P_{s}=0.3\times H\left(t-600\right)H\left(1400-t\right), \end{array}$$ (57)

where *H* is the Heaviside function. The corresponding IP_3_ timeseries and the resulting *C*_*cyt*_ are shown by the inset graph of Fig 3A and the blue curves in all panels, respectively. As shown in Fig 3A, *N* rapidly increased with the increase in *C*_*cyt*_ and slowly decreased to the baseline level, in contrast to the fast downstroke of *C*_*cyt*_. With a raised baseline level, the evolution of *N* during Ca^2+^ oscillations was analogous to the experimental observation described above. A similar pattern was observed in other mitochondrial variables. At the onset of the cytosolic Ca^2+^ spikes, the model generated rapid increases in *V*_*m*_, *ATP*_*c*_, and *ATP*_*m*_, followed by an extended period of decrease at a considerably slower rate than *C*_*cyt*_. Similar to the evolution of *N*, *V*_*m*_, *ATP*_*c*_, and *ATP*_*m*_ showed relatively slower dynamics than *C*_*cyt*_ (Fig 3B–3D). These model simulations are in agreement with the experimental data discussed above. We would like to point out that the evolution of *V*_*m*_ in the model is different from that of Wacquier et al. [22], which exhibits a transient decrease in the mitochondrial membrane potential at the onset of a cytosolic Ca^2+^ spike. However, the simultaneous increases of *C*_*cyt*_ and *V*_*m*_ are consistent with the experimental observations of Gaspers et al. [35, 36]. S2 Appendix discusses the opposing *V*_*m*_ behaviors between the Wacquier’s model and our model, and how they can be reconciled.

One caveat of this model is that it is inappropriate for studying how mitochondrial metabolic variables are correlated with obesity. More details are in Discussion and in S2 Appendix.

#### Ca^2+^ activities in MAMs

**Fig 4:**
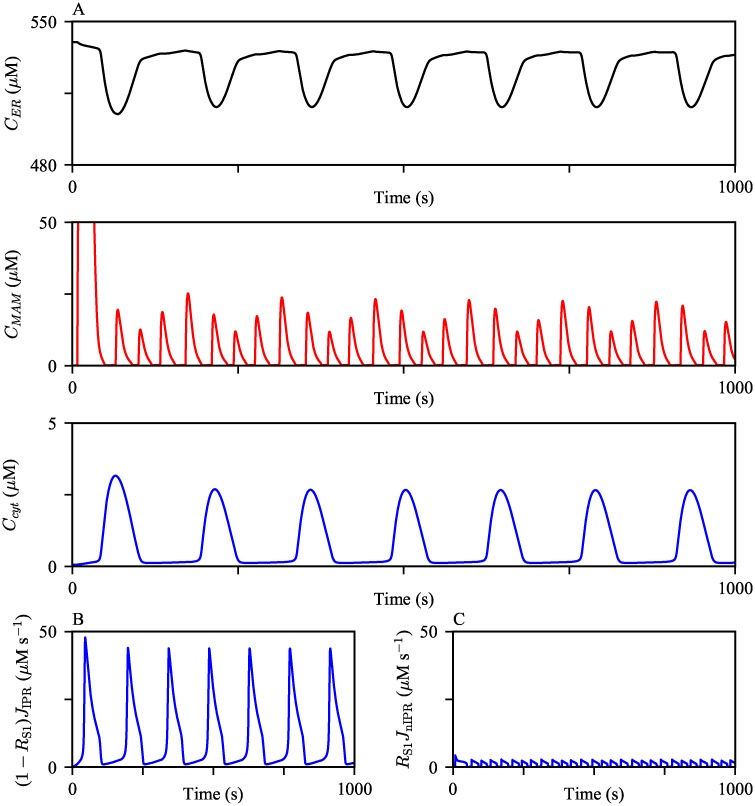
Ca^2+^ oscillations generated from the model exhibit varying orders of magnitude in different compartments. (A) The model was given continuous stimulation of IP_3_ with *P*_*s*_ = 0.3 *μ*M. From the top, the panels show Ca^2+^ oscillations in the ER, the MAM, and the bulk cytosol. (B and C) The magnitudes of IPR Ca^2+^ fluxes from the ER to the bulk cytosol and the MAM, respectively, during the oscillations shown in (A).

Experimental observations suggest that Ca^2+^ concentration in MAMs is about 5- to 10-fold higher than that of the cytosol, and about 10-fold lower than that in the ER lumen [38, 39]. We examined whether the model reproduces these phenomena. The model was continuously stimulated with a constant IP_3_ concentration at its steady state *P*_s_ = 0.3 *μ*M to generate oscillations in all three compartments (Fig 4A). The model simulations produced the expected order differences in the Ca^2+^ concentrations between the domains. We note that this model behavior is solely due to the model assumption of a significantly large volume difference between the cytosol and MAMs. Since the portion of the ER membrane that faces the cytosol is larger than the other section juxtaposing MAMs, it is not surprising to see the larger cytosolic IPR Ca^2+^ flux (Fig 4B and 4C). Nonetheless, the model simulated MAM Ca^2+^ oscillations with higher peaks, despite the relatively smaller MAM IPR Ca^2+^ fluxes.

**Fig 5:**
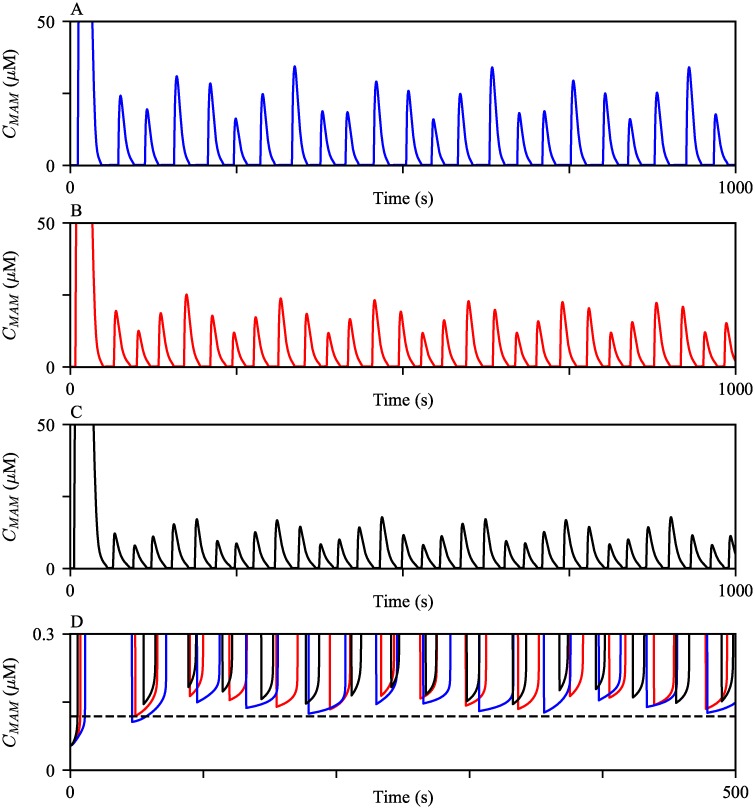
MAM Ca^2+^ oscillations generated with varying V_nSERCA_. The oscillations were generated with (A) *V*_nSERCA_ = 12 *μ*M s^−1^, (B) *V*_nSERCA_ = 10 *μ*M s^−1^, and (C) *V*_nSERCA_ = 8 *μ*M s^−1^. (D) Zoomed in at the basal level of MAM Ca^2+^ concentration after a spike, with different values of *V*_nSERCA_. The black dashed line indicates the cytosolic Ca^2+^ concentration right after a spike. For all three simulations, the model was given continuous stimulation of IP_3_ with *P*_*s*_ = 0.3 *μ*M.

Interestingly, the MAM Ca^2+^ activities are much more dynamic than those in the bulk cytosol, generating four MAM Ca^2+^ spikes per one spike in the bulk cytosol. We emphasize that the higher frequency of MAM Ca^2+^ oscillations is a model prediction that remains to be validated experimentally. There is no physiological explanation that suggests the levels of Ca^2+^ required for the activation and inhibition of the MAM IPR are different from those of the bulk cytosolic IPR. Thus, even a small Ca^2+^ flux into the MAM causes a large increase in the concentration, which quickly inhibits the MAM IPRs, generating Ca^2+^ spikes with a narrower width in MAMs. After a spike, MAM Ca^2+^ concentration returns to a lower level, but not lower than the level that a cytosolic Ca^2+^ spike returns to; see Fig 5D. This gives MAMs a better chance to induce another Ca^2+^ spike through Ca^2+^-induced Ca^2+^ release, shortening the interspike interval, and thus generating Ca^2+^ oscillations with a higher frequency than those in the cytosol. To confirm that a higher basal Ca^2+^ concentration is indeed an influential factor in the oscillation frequency difference between MAMs and the cytosol, we simulated oscillations with a larger and a smaller *V*_nSERCA_, which lowered and raised the base level of Ca^2+^, respectively. Fig 5A–5C shows the model simulations. As *V*_nSERCA_ decreased, both the average basal MAM Ca^2+^ concentration and the the frequency of MAM Ca^2+^ oscillations increased. We note that when we assumed no SERCA pumps in MAMs, i.e., *V*_nSERCA_ = 0 *μ*M s^−1^, the simulated Ca^2+^ oscillations in the cytosol and MAMs had the same oscillation frequency. However, the amplitude of the MAM Ca^2+^ oscillations was of the same order of magnitude as that of the cytosolic Ca^2+^ oscillations, which does not agree with the experimental observations that suggested much higher order of Ca^2+^ concentrations in MAMs than in the cytosol [38, 39].

**Fig 6:**
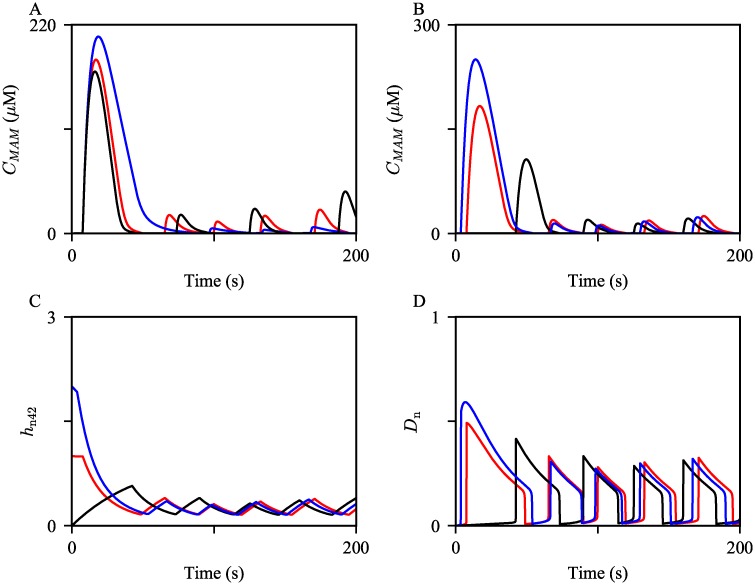
The first few MAM Ca^2+^ spikes generated with varying *H*_*IPR*_ and the initial condition of *h*_n42_. (A) The oscillations were simulated with *H*_*IPR*_ = 0.05 (blue), 0.1 (red), and 0.15 (black). (B) The oscillations were simulated with manually tuned *h*_n42_(0) = 2 (blue) and 0 (black). The red trajectory is the control, simulated with the steady state initial condition, *h*_n42_(0) = 0.99. (C) and (D) show the corresponding timeseries of *h*_n42_ and the fraction of MAM IPRs in the drive mode (*D*_n_), respectively, concurrently simulated with the trajectories shown in (B).

The first large MAM Ca^2+^ spike was generated mainly due to the slow rate of IPR inhibition by a high Ca^2+^ concentration, which is described by the parameter *H*_*IPR*_, and the initial condition of the variable *h*_n42_. We confirmed this through model simulations; the results are shown in Fig 6. When we introduced a faster rate of inhibition by increasing *H*_*IPR*_ to a larger value, the peak of the first MAM Ca^2+^ spike decreased; see the black curve in Fig 6A. Conversely, when *H*_*IPR*_ was decreased, the peak increased, shown by the blue curve in Fig 6A. As the inhibition rate gets slower, IPRs stay open for a longer time, allowing more Ca^2+^ to be released. Furthermore, the initial condition of the variable *h*_*n*42_ in Eq 13, which determines the transition rate *h*_n42_, also modulates the amplitude of the first MAM Ca^2+^ spike. The computed initial condition of *h*_*n*42_ was approximately 1. When we manually increased and decreased the initial condition to 2 and 0, the corresponding model simulations showed a larger and a smaller first MAM Ca^2+^ spike, respectively; see the blue and the black curves in Fig 6B. *h*_n42_ is positively correlated with the rate *q*_n42_, the transition rate at which MAM IPRs move from the park mode to the drive mode. Hence, a larger *h*_n42_ initial condition gives a faster transition rate from *t* = 0 till the onset of the first spike, as shown by Fig 6C. This allows the fraction of the MAM IPRs in the drive mode, *D*_n_, to be larger during the first spike, producing a bigger spike; see Fig 6D.

#### Control vs. obesity: Ca^2+^ transients

Arruda et al. [9] reported the effects of obesity on the morphology of hepatic ER and mitochondria. Their study involved lean mice and two different groups of obese mice, one that had been on high-fat diet (HFD) for 16 weeks, and the other, *ob*/*ob* mice. One of their main findings was that both groups of obese mice had a greater proportion of MAMs in liver cells. The authors also examined the expression levels of ER and mitochondrial proteins in liver lysates from the mice. Their western blot analysis showed that obese mice had higher expression of the IPR and PACS-2, an ER-mitochondrial tether protein. Interestingly, the expression level of MCU was higher in *ob*/*ob* mice, while the difference between lean and HFD mice was negligible. This suggests that the change in the expression of MCU may not occur at the early stages of obesity, although it is likely to be associated with obesity in the long-term.

Another part of their study traced intracellular Ca^2+^ activities, both in the cytosol and mitochondrial lumen. Liver cells from wild type and *ob*/*ob* mice were stimulated with ATP to induce subsequent Ca^2+^ releases from the ER. The results showed higher peaks of mitochondrial Ca^2+^ concentration in cells from obese mice, while there was no significant difference in the peaks of cytosolic Ca^2+^ concentrations. From this, the authors speculated that the higher mitochondrial Ca^2+^ peaks observed under obesity are related to having more ER-mitochondrial interactions in the cells, and consequently, increased direct Ca^2+^ transport through MAMs.

We do not know the exact orientation of Ca^2+^ channels in mouse hepatocytes. However, Arruda et al. [9] provide some guidelines for the relative expression levels between the control (lean) group and the obese group. According to Ref. [9], the MAM IPR expression level was almost doubled in *ob*/*ob* hepatocytes compared to wild type hepatocytes. Moreover, when they compared the IPR expression levels in liver lysates between the groups, hepatocytes from *ob*/*ob* mice showed a markedly higher level than those from the lean group. Although there was no direct comparison of the bulk cytosol IPR expression levels between the groups, we assumed that the bulk cytosol IPR expression level is also higher in the *ob*/*ob* group. Secondly, the *ob*/*ob* group showed a higher MCU expression level in the liver total lysates, compared to the lean group. Taking these experimental observations into account, we made some adjustments to the IPR and MCU Ca^2+^ fluxes to simulate Ca^2+^ dynamics in *ob*/*ob* hepatocytes. Furthermore, Fu et al. [40] reported that in obesity, SERCA pumps show impaired activity, causing the ER to be more leaky. Based on this, we increased $\bar{k}$ in Eqs 31 and 32, the parameter that determines the ‘leakiness’ of the ER, or the reverse power of SERCA. This parameter modification decreases the net SERCA flux, which is consistent with the findings of Fu et al. We emphasize that these parameter modifications are not the precise quantification of the protein levels. Rather, they are qualitative representations of the hepatocellular changes linked with obesity.

The parameters shown in Table 1 are control values, and when we simulated the model with this parameter set, which will be referred to as the *control model* from here on, model outcomes were regarded as the baseline behaviors for later comparison. Based on the experimental results discussed above, we modified some of the parameters to reflect cellular changes that are associated with obesity. Table 2 shows the parameter modifications. The model with the modified parameters will be referred to as the *obesity model*. We compared behaviors of the obesity model to those of the control model to confirm that the models correctly capture the difference between Ca^2+^ activities observed in hepatocytes from lean and obese mice.

**Table 2:** Modified parameters for the obesity model simulations.

Parameter	control model	obesity model
R_S1_	0.15	0.3
R_S2_	0.15	0.3
*k*_IPR_	0.3 s^−1^	0.35 s^−1^
*k*_nIPR_	0.15 s^−1^	0.3 s^−1^
V_MCU_	0.00001 *μ*M s^−1^	0.000013 *μ*M s^−1^
$\bar{k}$	1 × 10^−8^	1.25 × 10^−8^

**Fig 7:**
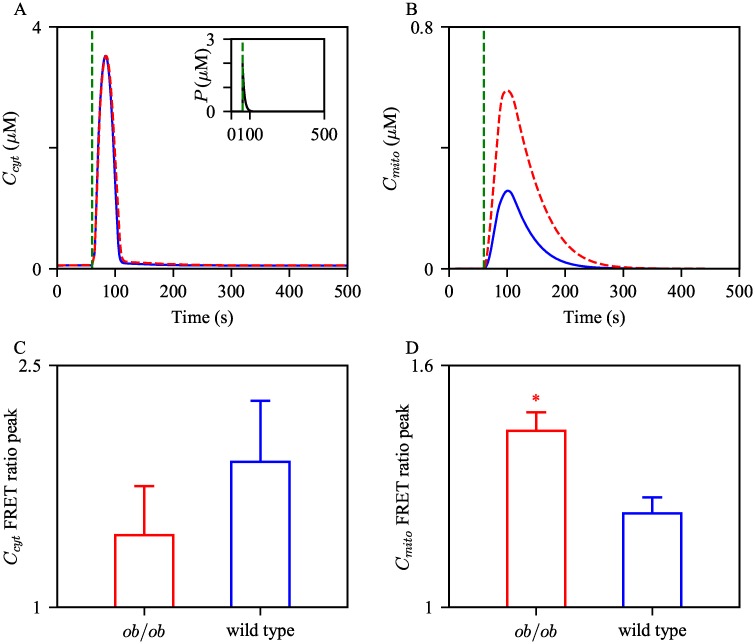
Effects of cellular changes associated with obesity on amplitudes of cytosolic and mitochondrial Ca^2+^ transients. The control model and the obesity model was given a pulse of IP_3_, Eq 55 with *M* = 10, *t*_0_ = 100, and Δ = 0.2, shown by the inset graph in (A). The cytosolic Ca^2+^ trajectories are shown in (A), while mitochondrial Ca^2+^ are shown in (B). The blue solid curves are the solutions from the control model, and the red dashed ones are from the obesity model. The green dashed vertical lines indicate the pulsing time. The bottom panels show the quantification of (C) cytosolic and (D) mitochondrial Ca^2+^ FRET ratio peaks in wild type and *ob*/*ob* hepatocytes. The experimental data were obtained from Arruda et al. [9]. * Student’s t-test *p*-value < 0.05.

Following the previous simulation setting, the control model and the obesity model were stimulated with a pulse of IP_3_. Fig 7A and 7B show the resulting Ca^2+^ timeseries in the cytosol and mitochondria, respectively, with the control parameter set and the adjusted set. Compared to the control model, the obesity model generated a mitochondrial Ca^2+^ transient with a higher peak, and a similar peak of cytosolic Ca^2+^ transient. These simulations are in agreement with the findings of Arruda et al. [9], shown by Fig 7C and 7D, which reported that the cytosolic Ca^2+^ transient FRET ratio peaks between the wild type and *ob*/*ob* hepatocytes show no significant difference, while the peak difference in mitochondria is statistically significant.

We then investigated the sensitivity of the peaks of cytosolic and mitochondrial Ca^2+^ transients to a small perturbation in each parameter, and how they are different between the two model conditions. Each parameter of interest was increased by 0.1%, and the subsequent changes in the peaks were recorded. Table 3 shows the percent change of each measurement induced from the percent change in parameters. For both models, the peaks are most sensitive to the change in *k*_IPR_, the IPR activity level in the bulk cytosol.

**Table 3:** Sensitivity of *C*_*cyt*_ and *C*_*mito*_ peaks to a small change in each parameter.

	control model		obesity model	
Parameter	$\frac{\%\Delta (C_{cyt}\;\text{peak})}{\%\Delta (\text{parameter})}$	$\frac{\%\Delta (C_{mito}\;\text{peak})}{\%\Delta (\text{parameter})}$	$\frac{\%\Delta (C_{cyt}\;\text{peak})}{\%\Delta (\text{parameter})}$	$\frac{\%\Delta (C_{mito}\;\text{peak})}{\%\Delta (\text{parameter})}$
R_S1_	-0.152	0.017	-0.403	-0.031
R_S2_	-0.002	0.156	-0.018	0.424
*k*_IPR_	2.11	2	1.91	0.811
*k*_nIPR_	-0.229	0.142	-0.032	0.373
V_MCU_	-0.052	0.785	-0.087	0.795
$\bar{k}$	-0.609	-0.732	-0.583	-0.406

### Model predictions

**Fig 8:**
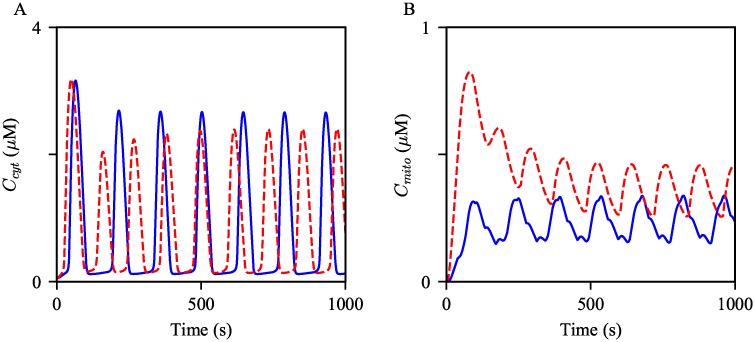
Effects of the cellular changes associated obesity on Ca^2+^ oscillations. Oscillations of (A) cytosolic and (B) mitochondrial Ca^2+^ concentrations generated from the model with different parameter sets. The blue oscillations were generated from the model with the control parameters. The red dashed oscillations were generated with the modified parameters as in Table 2. The model was given continuous stimulation of IP_3_ with *P*_s_ = 0.3 *μ*M.

The panels in Fig 8 show Ca^2+^ oscillations generated from the control and obesity models with the same magnitude of stimulation. The simulations suggest that hepatocytes from obese mice may exhibit faster Ca^2+^ oscillations compared to cells from lean mice. To quantify the frequency difference between the model, we simulated the solutions for two hours of simulation time, then computed the average frequency for the comparison. The average frequency of the oscillations in the obesity model was about 19% higher than that in the control model. Moreover, mitochondrial Ca^2+^ oscillations in the obesity model showed about 86% higher average level than those in the control model. In the following sections, we scrutinize how each cellular change associated with obesity modulates Ca^2+^ oscillations.

#### Increased MAM formation

**Fig 9:**
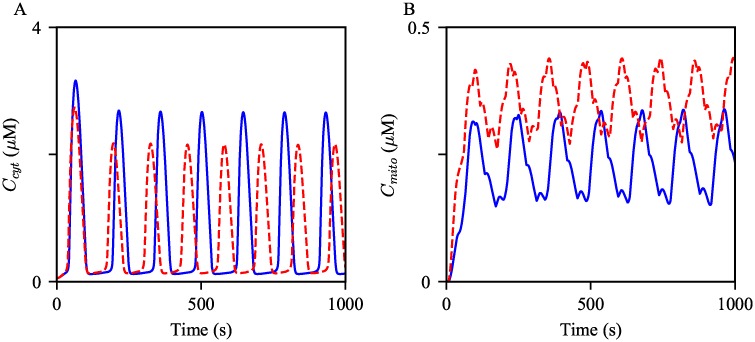
Effects of increased MAMs on Ca^2+^ oscillations. Oscillations of (A) cytosolic and (B) mitochondrial Ca^2+^ concentrations generated from the control model, in blue, and the model with the increased *R*_S_’s, in red dashed. The model was given continuous stimulation of IP_3_ with *P*_*s*_ = 0.3 *μ*M.

It is already evident in Fig 2 that an increase in the degree of MAM formation induces an increase in the peak of a mitochondrial Ca^2+^ transient. This section probes the effects of the upregulation of MAM formation on Ca^2+^ oscillations. Fig 9 shows two sets of the Ca^2+^ oscillations generated from the control model, and the model with the increased *R*_S1_ and *R*_S2_. The model results suggest a positive correlation between the degree of MAM formation and the oscillation frequency. Interestingly, the average peak of cytosolic Ca^2+^ oscillations was decreased, while that of mitochondrial Ca^2+^ oscillations was increased. We have previously discussed that a possible mechanism underlying the amplitude change in mitochondrial Ca^2+^ activities associated with the enhancement of MAM formation is a synergistic process that comes from the MCU’s low Ca^2+^ affinity and the small volume of MAMs. As stated in the introduction, cells tightly regulate frequencies and amplitudes of Ca^2+^ oscillations to orchestrate many cellular activities. Thus, although the magnitude of the frequency change is small, its effect on cellular homeostasis may be significant.

#### Increased IPR activity

**Fig 10:**
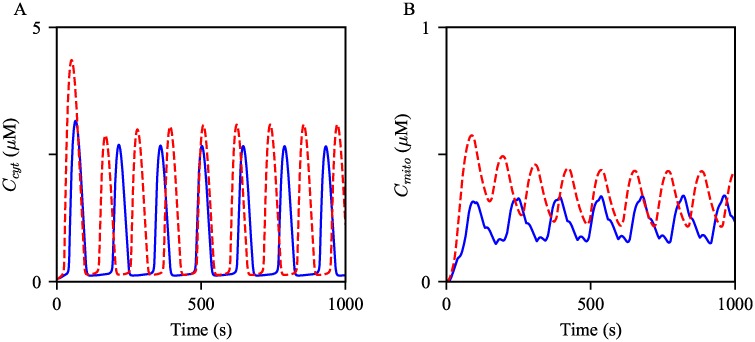
Effects of increased IPR activity on Ca^2+^ oscillations. Oscillations of (A) cytosolic and (B) mitochondrial Ca^2+^ concentrations generated from the control model, in blue, and the model with the increased *k*_IPR_ and *k*_nIPR_, in red dashed. The model was given continuous stimulation of IP_3_ with *P*_s_ = 0.3 *μ*M.

Secondly, we simulated Ca^2+^ oscillations with increased *k*_IPR_ and *k*_nIPR_ to evaluate the effects of increased IPR activity on Ca^2+^ oscillations. Such parameter adjustments ultimately lead to an increase in the magnitude of total Ca^2+^ flux through the IPR, either from an increased number of channels or an increase in the maximum strength of each channel. Thus, we expected that the increased IPR parameters would generate Ca^2+^ oscillations with larger amplitudes in both the cytosol and mitochondria. The results are shown in Fig 10, supporting our conjecture. An increase in the oscillation frequency was another noticeable response to the parameter changes.

#### Increased MCU activity

**Fig 11:**
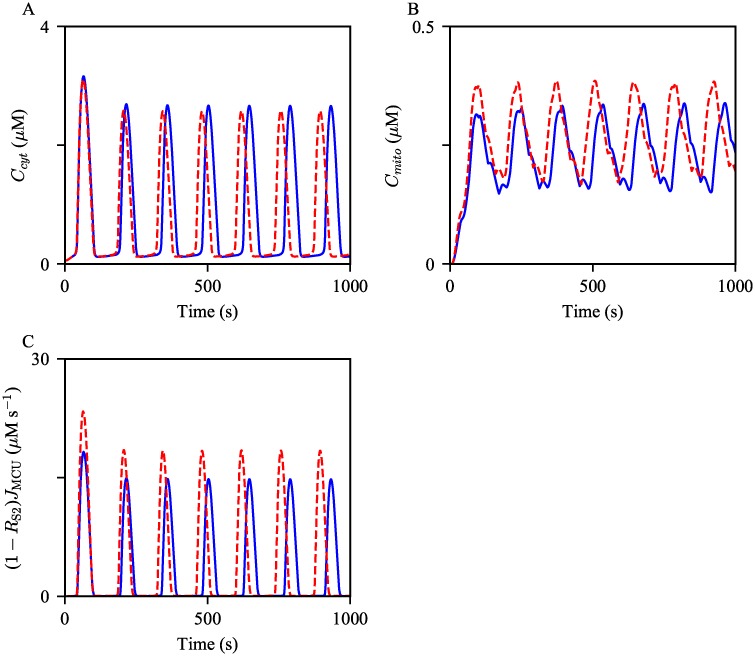
Effects of increased MCU activity on Ca^2+^ oscillations. Oscillations of (A) cytosolic, (B) mitochondrial Ca^2+^ concentrations generated from the control model, in blue, and with the increased *V*_MCU_, in red dashed. The corresponding Ca^2+^ fluxes from the cytosol to mitochondria are shown in (C). The model was given continuous stimulation of IP_3_ with *P*_s_ = 0.3 *μ*M.

This section discusses the effects of an increase in MCU activity level on cytosolic and mitochondrial Ca^2+^ oscillations. The model simulations are shown in Fig 11. Analogous to the above model results, the increased parameters had a positive effect on the oscillation frequency. Moreover, the average amplitude of Ca^2+^ oscillations in the cytosol showed a small decrease, while that in mitochondria was moderately increased. The most probable explanation for the amplitude changes is that the enhanced activity of MCUs transports a larger amount of Ca^2+^ from the cytosol to mitochondria, and the magnitude of change is more substantial in mitochondria than that in the cytosol due to the compartments’ volume difference. This is evident in Fig 11C, which shows a higher rate of MCU Ca^2+^ flux from the cytosol per Ca^2+^ spike, in association of the parameter increase.

#### Decreased net SERCA flux

Lastly, we studied how compromised SERCA activity effects Ca^2+^ oscillations. As explained in Materials and Models, the parameter $\bar{k}$ defines the degree of the reverse reaction of SERCA. Thus, an increase in $\bar{k}$ would result in a decrease in the net SERCA flux, and subsequently, a reduction in the ER Ca^2+^ concentration. At the steady state, i.e., when *P*_s_ = 0 *μ*M, the SERCA Ca^2+^ fluxes are zero. We can derive the following relationships by solving the algebraic equation *J*_SERCA_ = 0 for $\bar{k}$:

$$\begin{array}{c} \bar{k}=\frac{C_{cyt}^{2}}{C_{ER}^{2}} \end{array}$$ (58)

**Fig 12:**
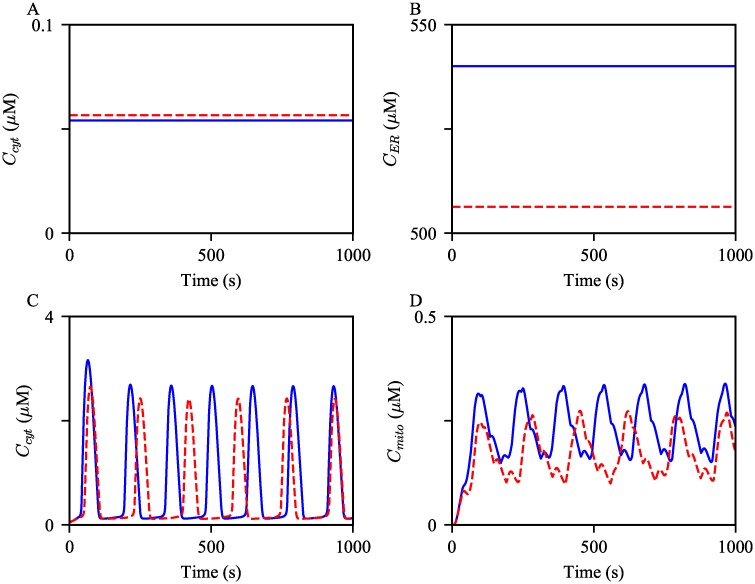
Effects of decreased net SERCA flux on the steady state Ca^2+^ concentrations and Ca^2+^ oscillations. Ca^2+^ concentrations in (A) the cytosol and (B) the ER at the steady state, *P*_s_ = 0 *μ*M, simulated from the control model (in blue) and the model with increased $\bar{k}$ (in red). Oscillations of (C) cytosolic, (D) mitochondrial Ca^2+^ concentrations generated from the control model, in blue, and with the increased $\bar{k}$, in red dashed. The model was given continuous stimulation of IP_3_ with *P*_s_ = 0.3 *μ*M.

Therefore, as $\bar{k}$ increases, the steady state ER Ca^2+^ concentration is expected to decrease, while the cytosolic Ca^2+^ concentration increases. The model simulations showed the expected concentration changes, see Fig 12A and 12B. As there is less Ca^2+^ stored in the ER, less Ca^2+^ is released through the IPRs. This is corroborated by the simulations shown in Fig 12C and 12D; the resulting oscillations with the decreased $\bar{k}$ have lower amplitudes.

#### Ca^2+^ oscillations with obesity

**Fig 13:**
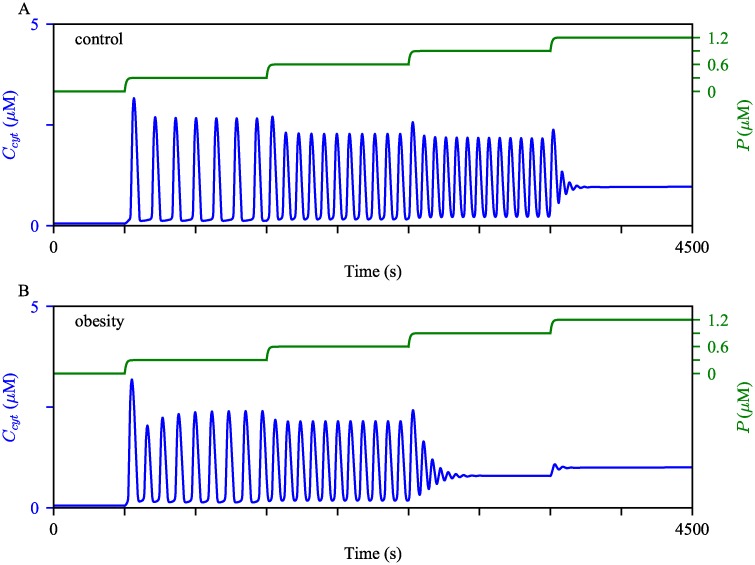
Robustness of Ca^2+^ oscillations under different model conditions. We perturbed (A) the control model and (B) the obesity model with gradually increasing stimulation. Initially, *P*_s_ was at 0 *μ*M, then was increased to 0.3 *μ*M, 0.6 *μ*M, 0.9 *μ*M, and then to 1.2 *μ*M at *t* = 500 s, 1500 s, 2500 s, and 3500 s, respectively. The cytosolic Ca^2+^ concentrations are shown in blue, with the scale on the left y-axis. The green timeseries represent the IP_3_ concentration, with the scale on the right y-axis.

We postulate that liver cells from different health conditions have varying thresholds for the cessation of their Ca^2+^ oscillations. For each parameter set, the model was given continuous stimulation with five distinct regimes where *P*_s_ is incrementally adjusted, as shown by the green curves in the panels of Fig 13. It is clear that the obesity model reached the cessation of Ca^2+^ oscillations at a lower concentration of IP_3_, compared to the control model.

To verify these predictions, we suggest measuring Ca^2+^ responses in liver cells with each condition to a wide range of IP_3_ concentration, and see if the average IP_3_ oscillatory range is decreased in the *ob*/*ob* condition. However, one of the challenges of this experiment is that, due to cell-to-cell variability, it is critical to measure responses from a population of cells before drawing a conclusion.

## Discussion

We have presented a mathematical model for Ca^2+^ dynamics in mouse hepatocytes. To our knowledge, this model is the first mathematical model to explicitly express Ca^2+^ concentration in MAMs as a dynamical variable and also show MAM Ca^2+^ levels to be within reasonable proportions of the Ca^2+^ levels in the other domains. The first aim of the model was to reproduce the data reported by Arruda et al. where hepatocytes with more MAMs exhibited ATP-induced Ca^2+^ transient with higher peaks in mitochondrial Ca^2+^ concentration, and hepatocytes from obese animals generated higher peaks of mitochondrial Ca^2+^ transient, compared to cells from lean animals, while having no significant difference in the peaks of cytosolic Ca^2+^ transient [9].

Arruda et al. also compared hepatic cellular characteristics between different groups of mice. They had a group of lean mice as their control, and two groups of mice, one that had been under high fat diet (HFD) and the other genetically obese (*ob*/*ob*) mice, for mouse models of obesity. The *ob*/*ob* mouse cells showed higher expression levels of IPR and MCU, as well as a higher degree of MAM formation.

We used the model to study how Ca^2+^ signals are altered by the cellular change associated with obesity. According to model simulations, hepatocytes from obese animals exhibit faster Ca^2+^ oscillations than those from healthy animals. Moreover, the average mitochondrial Ca^2+^ concentration is higher under obesity.

Metabolic flexibility refers to the ability of the organism to adapt its fuel source, depending on availability and need [41], and emerging evidence suggests the involvement of MAMs in this adaptation [42]. Interestingly, Rieusset et al. [43] reported a link between the disruption of ER-mitochondrial Ca^2+^ exchange and hepatic insulin resistance in their mouse model. As we have shown in this paper, hepatic cellular changes associated with obesity affect Ca^2+^ oscillation frequencies and amplitudes. However, the question of whether the altered Ca^2+^ dynamics plays a causal role in the development of hepatic insulin resistance and metabolic diseases remains to be explored. We hope that our model can help in addressing such puzzles.

### Plasma membrane Ca^2+^ fluxes under obesity

Our model is an open-cell type that exhibits dynamic total Ca^2+^ concentration (*C*_*t*_), which is determined by Ca^2+^ fluxes across the plasma membrane. Though we have not introduced any changes to these fluxes for model simulations of the obesity condition, there is experimental evidence that suggests otherwise. Arruda et al. [44] showed diminished protein-protein interaction between the ER membrane and the plasma membrane that, upon ER Ca^2+^ depletion, facilitates store-operated Ca^2+^ entry (SOCE). Such protein-protein interactions form another type of Ca^2+^ microdomain, and they are not explicitly included in the model as they are not the main subject of this paper. However, a follow-up theoretical study of these ER-plasma membrane junctions in a whole cell context, similar to our ER-mitochondria contact model, would be relevant and potentially useful in gaining a complete understanding.

### Model limitations

The model is deterministic, which assumes synchronized behaviors of the activated IPRs, within each compartment. This allows relatively fast simulations of Ca^2+^ oscillations (periodic solutions). Furthermore, as in many previous mathematical models of Ca^2+^ dynamics [18, 22, 26, 28], the model assumes a homogeneous Ca^2+^ profile throughout the bulk cytosol. These underlying assumptions are physiologically inaccurate, as many experimental studies show the stochastic nature of intracellular Ca^2+^ activities and clustered spatial distributions of Ca^2+^ channels that regulate such activities. Nevertheless, Cao et al. [24] demonstrated that IPR stochasticity is not pivotal for qualitative predictions of oscillation traits, such as frequency. Furthermore, Voorsluijs et al. [45] presented a heuristic model that can explain the coexistence of subcellular Ca^2+^ signals that occur due to the intrinsically stochastic nature of the IPR activities, and the cell level global Ca^2+^ spikes that are more likely to arise from a deterministic mechanism. The authors showed that the oscillation periods generated from a deterministic version of their model lie within the interspike interval distributions simulated from a stochastic version of the model. Thus, we find it reasonable to utilize a deterministic model, such as ours, to simulate and probe the attributes of Ca^2+^ activities, at least for those at the cell level.

The model is not applicable for studying the effects of obesity and obesity-related changes in Ca^2+^ dynamics on mitochondrial metabolism. For the obesity model simulations, we only modified the parameters that are directly associated with Ca^2+^ dynamics. This is not to diminish the criticality of mitochondrial metabolic mechanisms nor to suggest that there is no effect of obesity on the mechanisms. There certainly is a plethora of experimental evidence that suggests correlations between obesity and mitochondrial dysfunction that range from structural functions such as fusion and fission to biochemical functions. What we do not fully comprehend yet is, which parts of the pathways are linked with obesity, and how they communicate with each other. Furthermore, the model is not comprehensive enough to describe mitochondrial metabolic pathways under different cell conditions. For instance, the process of glycolysis is represented by a single parameter, *k*_gly_, which is an over simplification of the process that is highly correlated with the nutrient availability, i.e., the cell’s metabolic state. Admittedly, the current state of the model and assumptions is not suitable for investigating possible mechanisms underlying the adverse effects of obesity on mitochondrial metabolic dynamics.

The best mathematical model for describing cellular Ca^2+^ dynamics would be a system of stochastic partial differential equations with spatial and temporal discretization. However, there is a trade-off between sufficient accuracy, computational efficiency, and data availability.

## Supporting information

S1 File .ode file for simulating the model solutions. (ODE)

S1 Appendix Non-dimensionalization and reduction of the full model. (PDF)

S2 Appendix Supplementary figures. (PDF)
