# Supplementary material for: A mathematical model of calcium dynamics: Obesity and mitochondria-associated ER membranes
Source: PLoS Comput Biol. 2019 Aug 22;15(8):e1006661. doi: 10.1371/journal.pcbi.1006661 (PMC6726250; doi:10.1371/journal.pcbi.1006661)
Supplement: S1 Appendix — (PDF) [file pcbi.1006661.s002.pdf]

S1 Appendix

The full model consists of fifteen variables:

$$\frac{d}{dt}C_{cyt} = f_c\Bigg[(1 - R_{\mathrm{S1}})(J_{\mathrm{IPR}} - J_{\mathrm{SERCA}}) + \frac{1 - R_{\mathrm{S2}}}{R_{\mathrm{V3}}}(J_{\mathrm{NCX}} - J_{\mathrm{MCU}}) + J_{\mathrm{diff}} + J_{\mathrm{in}} - J_{\mathrm{pm}}\Bigg] \tag{1}$$

$$\frac{d}{dt}C_{MAM} = f_c\Bigg[R_{\mathrm{V1}}R_{\mathrm{S1}}(J_{\mathrm{nIPR}} - J_{\mathrm{nSERCA}}) + \frac{R_{\mathrm{V1}}R_{\mathrm{S2}}}{R_{\mathrm{V3}}}(J_{\mathrm{nNCX}} - J_{\mathrm{nMCU}}) - R_{\mathrm{V1}}J_{\mathrm{diff}}\Bigg] \tag{2}$$

$$\frac{d}{dt}C_{mito} = f_m\Bigg[R_{\mathrm{S2}}(J_{\mathrm{nMCU}} - J_{\mathrm{nNCX}}) + (1 - R_{\mathrm{S2}})(J_{\mathrm{MCU}} - J_{\mathrm{NCX}})\Bigg] \tag{3}$$

$$\frac{d}{dt}C_t = J_{\mathrm{in}} - J_{\mathrm{pm}} \tag{4}$$

$$\frac{d}{dt}P = \tau_p(P_{\mathrm{s}} - P) + \mathrm{pulse} \tag{5}$$

$$\frac{d}{d\hat{t}}h_{42} = \lambda_{h_{42}}(h_{42}^{\infty} - h_{42}) \tag{6}$$

$$\frac{d}{d\hat{t}}h_{\mathrm{n42}} = \lambda_{h_{n42}}(h_{\mathrm{n42}}^{\infty} - h_{\mathrm{n42}}) \tag{7}$$

$$\frac{d}{d\hat{t}}m_{42} = \lambda_{m_{42}}(m_{42}^{\infty} - m_{42}) \tag{8}$$

$$\frac{d}{d\hat{t}}m_{\mathrm{n42}} = \lambda_{m_{42}}(m_{\mathrm{n42}}^{\infty} - m_{\mathrm{n42}}) \tag{9}$$

$$\frac{d}{d\hat{t}}h_{24} = \lambda_{h_{24}}(h_{42}^{\infty} - h_{42}) \tag{10}$$

$$\frac{d}{d\hat{t}}m_{24} = \lambda_{m_{24}}(m_{42}^{\infty} - m_{42}) \tag{11}$$

$$\frac{d}{dt}ADP_c = I_{\mathrm{hyd}} - \frac{I_{\mathrm{ant}}}{R_{\mathrm{V3}}} \tag{12}$$

$$\frac{d}{dt}ADP_m = I_{\mathrm{ant}} - I_{\mathrm{F1FO}} \tag{13}$$

$$\frac{d}{dt}N = I_{\mathrm{pdh}} - I_{\mathrm{o}} + I_{\mathrm{agc}} \tag{14}$$

$$\frac{d}{dt}V_m = \frac{1}{C_p}(a_1 I_{\mathrm{o}} - a_2 I_{\mathrm{F1FO}} - I_{\mathrm{ant}} - I_{\mathrm{Hleak}} - (1 - R_{\mathrm{S2}})(J_{\mathrm{NCX}} + 2J_{\mathrm{MCU}}) - R_{\mathrm{S2}}(J_{\mathrm{nNCX}} + 2J_{\mathrm{nMCU}}) - I_{\mathrm{agc}}) \tag{15}$$

To non-dimensionalize the model, we first redefined the model variables with dimensionless variables,

$$\begin{aligned}
C_{cyt} &= Q_{cyt} \cdot \widehat{C_{cyt}}, & C_{MAM} &= Q_{MAM} \cdot \widehat{C_{MAM}} \\
C_{mito} &= Q_{mito} \cdot \widehat{C_{mito}}, & C_t &= Q_t \cdot \widehat{C_t} \\
P &= Q_p \cdot \widehat{P}, & ADP_m &= Q_{Am} \cdot \widehat{ADP_m}, \\
ADP_c &= Q_{Ac} \cdot \widehat{ADP_c}, & N &= Q_N \cdot \widehat{N}, \\
V_m &= Q_{Vm} \cdot \widehat{V_m}, & t &= T \cdot \widehat{t},
\end{aligned} \tag{16}$$

and dimensionless fluxes and rates

$$\begin{aligned}
\bar{J}_\star &= \frac{J_\star}{V_{\text{nSERCA}}} && \text{for } \star = \text{IPR, nIPR, SERCA, nSERCA, diff,} \\
& && \quad \text{MCU, nMCU, NCX, nNCX} \\
\bar{J}_\dagger &= \frac{V_{\text{SOCC}} J_\dagger}{V_{\text{pm}}^2} && \text{for } \dagger = \text{leakin, SOCC, ROCC, pm} \\
\bar{I}_\triangle &= \frac{I_\triangle}{V_{\text{nSERCA}}} && \text{for } \triangle = \text{hyd, ant, F1FO, pdh, o, agc, Hleak}
\end{aligned} \tag{17}$$

Then we derived the following dimensionless version of the system:

$$\begin{aligned}
\frac{d}{d\hat{t}}\widehat{C_{cyt}} = \frac{T V_{\text{nSERCA}} f_c R_{\text{V2}}}{Q_{cyt} R_{\text{V3}}} \Bigg[& \frac{R_{\text{V3}}}{R_{\text{V2}}}(1 - R_{\text{S1}})(\bar{J}_{\text{IPR}} - \bar{J}_{\text{SERCA}}) \\
& + \frac{1}{R_{\text{V2}}}(1 - R_{\text{S2}})(\bar{J}_{\text{NCX}} - \bar{J}_{\text{MCU}}) + \frac{R_{\text{V3}}}{R_{\text{V2}}}\bar{J}_{\text{diff}} \Bigg] + \frac{T V_{\text{pm}}^2 f_c}{Q_{cyt} V_{\text{SOCC}}} \left[\bar{J}_{\text{in}} - \bar{J}_{\text{pm}} \right]
\end{aligned} \tag{18}$$

$$\begin{aligned}
\frac{d}{d\hat{t}}\widehat{C_{MAM}} = \frac{T V_{\text{nSERCA}} f_c R_{\text{V1}}}{Q_{MAM} R_{\text{V3}}} \Bigg[& R_{\text{V3}} R_{\text{S1}}(\bar{J}_{\text{nIPR}} - \bar{J}_{\text{nSERCA}}) \\
& + R_{\text{S2}}(\bar{J}_{\text{nNCX}} - \bar{J}_{\text{nMCU}}) - R_{\text{V3}}\bar{J}_{\text{diff}} \Bigg]
\end{aligned} \tag{19}$$

$$\frac{d}{d\hat{t}}\widehat{C_{mito}} = \frac{T V_{\text{nSERCA}} f_m R_{\text{V2}}}{Q_{mito} R_{\text{V3}}} \left[\frac{R_{\text{V3}}}{R_{\text{V2}}} R_{\text{S2}}(\bar{J}_{\text{nMCU}} - \bar{J}_{\text{nNCX}}) + \frac{R_{\text{V3}}}{R_{\text{V2}}}(1 - R_{\text{S2}})(\bar{J}_{\text{MCU}} - \bar{J}_{\text{NCX}}) \right] \tag{20}$$

$$\frac{d}{d\hat{t}}\widehat{C_t} = \frac{T V_{\text{pm}}^2}{Q_t V_{\text{SOCC}}} \left[\bar{J}_{\text{in}} - \bar{J}_{\text{pm}} \right] \tag{21}$$

$$\frac{d}{d\hat{t}}\widehat{P} = T\tau_p\left[\frac{P_{\rm s}}{Q_p} - \widehat{P}\right] + \text{pulse} \tag{22}$$

$$\frac{d}{d\hat{t}}h_{42} = T\lambda_{h_{42}}\left[h_{42}^{\infty} - h_{42}\right] \tag{23}$$

$$\frac{d}{d\hat{t}}h_{\rm n42} = T\lambda_{h_{n42}}\left[h_{\rm n42}^{\infty} - h_{\rm n42}\right] \tag{24}$$

$$\frac{d}{d\hat{t}}m_{42} = T\lambda_{m_{42}}\left[m_{42}^{\infty} - m_{42}\right] \tag{25}$$

$$\frac{d}{d\hat{t}}m_{\rm n42} = T\lambda_{m_{42}}\left[m_{\rm n42}^{\infty} - m_{\rm n42}\right] \tag{26}$$

$$\frac{d}{d\hat{t}}h_{24} = T\lambda_{h_{24}}\left[h_{24}^{\infty} - h_{24}\right] \tag{27}$$

$$\frac{d}{d\hat{t}}m_{24} = T\lambda_{m_{24}}\left[m_{24}^{\infty} - m_{24}\right] \tag{28}$$

$$\frac{d}{d\hat{t}}\widehat{ADP_c} = \frac{TV_{\rm nSERCA}}{Q_{Ac}R_{\rm V2}}\left[R_{\rm V2}\bar{I}_{\rm hyd} - R_{\rm V2}\bar{I}_{\rm ant}/R_{\rm V3}\right] \tag{29}$$

$$\frac{d}{d\hat{t}}\widehat{ADP_m} = \frac{TV_{\rm nSERCA}}{Q_{Am}}\left[\bar{I}_{\rm ant} - \bar{I}_{\rm F1FO}\right] \tag{30}$$

$$\frac{d}{d\hat{t}}\widehat{N} = \frac{TV_{\rm nSERCA}}{Q_N R_{\rm V2}}\left[R_{\rm V2}\bar{I}_{\rm pdh} - R_{\rm V2}\bar{I}_{\rm o} + R_{\rm V2}\bar{I}_{\rm agc}\right] \tag{31}$$

$$\begin{aligned}\frac{d}{d\hat{t}}\widehat{V_m} = \frac{TV_{\rm nSERCA}}{C_pQ_VR_{\rm V2}R_{\rm V3}}\Big[&R_{\rm V2}R_{\rm V3}a_1\bar{I}_{\rm o} - R_{\rm V2}R_{\rm V3}a_2\bar{I}_{\rm F1FO} - R_{\rm V2}R_{\rm V3}\bar{I}_{\rm ant} - R_{\rm V2}R_{\rm V3}\bar{I}_{\rm Hleak}\\ &- R_{\rm V2}R_{\rm V3}(1-R_{\rm S2})(\bar{J}_{\rm NCX} + 2\bar{J}_{\rm MCU}) - R_{\rm V2}R_{\rm V3}R_{\rm S2}(\bar{J}_{\rm nNCX} + 2\bar{J}_{\rm nMCU})\\ &- R_{\rm V2}R_{\rm V3}\bar{I}_{\rm agc}\Big]\end{aligned} \tag{32}$$

For every equation, the term in $\left[\cdots\right]$ is approximately of order $O(1)$. The gating variable rates are $\lambda_{m_{42}} = \lambda_{m_{24}} = 100\ \text{s}^{-1}$ and $\lambda_{h_{24}} = 40\ \text{s}^{-1}$. These rates are estimated directly from dynamical single channel data [1, 2].

We chose $T = Q_{cyt}/(V_{\rm nSERCA}f_c)$. Based on numerical simulations of the model, typical concentration scale for the ions are of orders $Q_{cyt} = 1\ \mu$M, $Q_{MAM} = 10\ \mu$M, $Q_{mito} = 0.1\ \mu$M, $Q_t = 10^3$ μM, $Q_{Ac} = 10^3\ \mu$M, $Q_{Am} = 10^4\ \mu$M, $Q_N = 10^2\ \mu$M. A typical scale for mitochondrial membrane potential is $Q_V = 10^2$ mV. With these scales, the magnitudes of the right hand sides of the equations

Eq. (18) – Eq. (32) are:

$$\begin{aligned}
&\frac{d}{d\hat{t}}\widehat{C_{cyt}} \sim O(0.1) && \frac{d}{d\hat{t}}\widehat{C_{MAM}} \sim O(10) && \frac{d}{d\hat{t}}\widehat{C_{mito}} \sim O(0.1)\\
&\frac{d}{d\hat{t}}\widehat{C_t} \sim O(0.0001) && \frac{d}{d\hat{t}}\widehat{P} \sim O(1) && \\
&\frac{d}{d\hat{t}}h_{42} \sim O(0.1) && \frac{d}{d\hat{t}}h_{\mathrm{n}42} \sim O(0.1) && \frac{d}{d\hat{t}}m_{42} \sim O(1000)\\
&\frac{d}{d\hat{t}}m_{\mathrm{n}42} \sim O(1000) && \frac{d}{d\hat{t}}h_{24} \sim O(100) && \frac{d}{d\hat{t}}m_{24} \sim O(1000)\\
&\frac{d}{d\hat{t}}\widehat{ADP_c} \sim O(0.01) && \frac{d}{d\hat{t}}\widehat{ADP_m} \sim O(0.01) && \\
&\frac{d}{d\hat{t}}\widehat{N} \sim O(0.1) && \frac{d}{d\hat{t}}\widehat{V_m} \sim O(0.001) &&
\end{aligned} \tag{33}$$

The model contains multiple timescales with the IPR gating variables evolving on the fastest and $\widehat{C_t}$ on the slowest. Using the quasi-steady-state approximation, we assumed that the gating variables reach their quasi-equilibria instantaneously. We note that this model reduction method is not well established for systems with multiple timescales. However, we compared model simulations of the full model with those of the reduced model, and confirmed that the dynamical characteristics are preserved under the reduction. Thus, we find that the quasi-steady-state reduction was suitable for our Ca^{2+} model.

References

1. Mak DOD, Pearson JE, Loong KPC, Datta S, Fernández-Mongil M, Foskett JK. Rapid ligand-regulated gating kinetics of single inositol 1,4,5-trisphosphate receptor Ca^{2+} release channels. EMBO reports. 2007;**8**(11):1044–1051.

2. Cao P, Tan X, Donovan G, Sanderson MJ, Sneyd J. A deterministic model predicts the properties of stochastic calcium oscillations in airway smooth muscle cells. PLoS Comput Biol.

2014;10(8):e1003783.
