# Supplementary material for: A mathematical model of calcium dynamics: Obesity and mitochondria-associated ER membranes
Source: PLoS Comput Biol. 2019 Aug 22;15(8):e1006661. doi: 10.1371/journal.pcbi.1006661 (PMC6726250; doi:10.1371/journal.pcbi.1006661)
Supplement: S2 Appendix — (PDF) [file pcbi.1006661.s003.pdf]

S2 Appendix

Wacquier model vs. Han-Periwal model: Mitochondrial membrane potential

As pointed out in the main manuscript, the mitochondrial membrane potential, V_m, in our model behaves differently from that of the model presented by Wacquier et al. [1]. In our model, the onset of a mitochondrial Ca^{2+} spike was accompanied by an increase in V_m, whereas in the Wacquier model, V_m showed a transient decrease. This was very puzzling to us as the equations for describing the dynamics of the mitochondrial variables were the same, and only a few parameters were adjusted. To understand this discrepancy between the models, we tested which of the modified parameters was responsible.

By checking the parameters one by one, we found the parameter of interest. When we decreased V_{MCU}, which represent the level of MCU activity, in the Wacquier model from its original value, 0.0006, to 0.00001, the simulated V_m oscillations behaved like the ones in our model, with an increase at the onset of a mitochondrial Ca^{2+} spike (see Figure 1A and 1B). Moreover, when we increased V_{MCU} to 0.0001 in our model, the simulated V_m showed a transient decrease at the onset of a Ca^{2+} spike (see Figure 1C and 1D). These findings suggest that there is no fundamental difference between the dynamics of the models, and that V_{MCU} just needs to be large enough to induce a significant drop in V_m.

Effects of obesity on mitochondrial metabolism

As discussed in the main text, this model is not detailed enough to study the effects of obesity on the mitochondrial metabolic variables. Figure 2 shows the simulated mitochondrial variables with the control parameter set and the obesity parameter set. The trajectories do not vary much between the parameter sets. However, we highly doubt that this is plausible, as there is experimental evidence that links mitochondrial dysfunction with obesity.

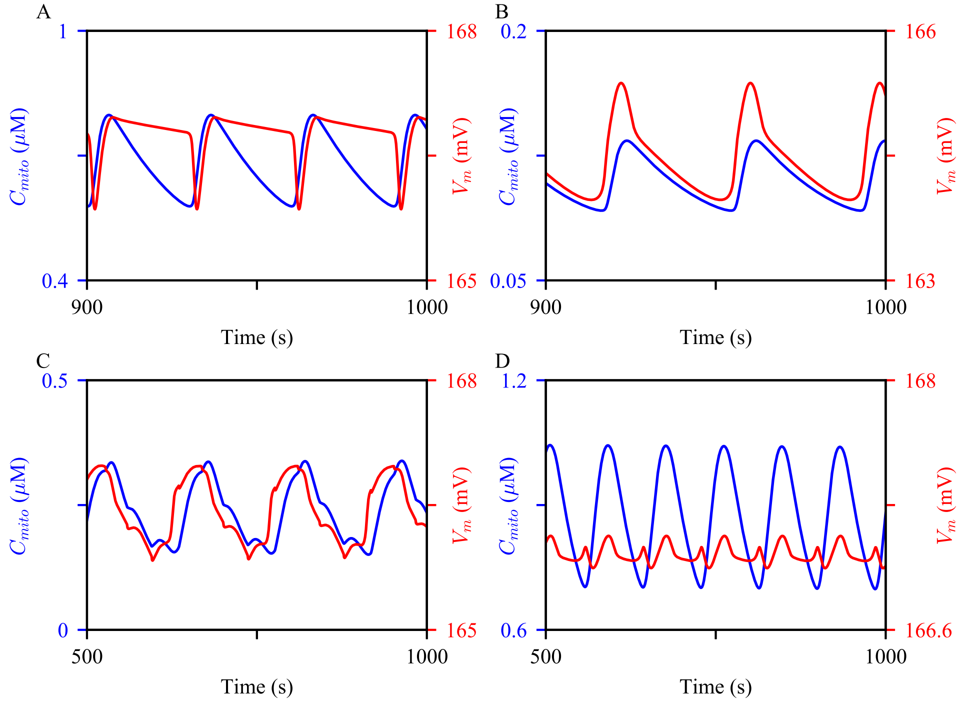

Figure 1: Mitochondrial Ca^{2+} (in blue) and membrane potential (in red) oscillations generated from the Wacquier model with (A) $V_{\mathrm{MCU}} = 0.0006\ \mu\mathrm{M\ s}^{-1}$ and (B) $V_{\mathrm{MCU}} = 0.00001\ \mu\mathrm{M\ s}^{-1}$, and from our model with (C) $V_{\mathrm{MCU}} = 0.00001\ \mu\mathrm{M\ s}^{-1}$ and (D) $V_{\mathrm{MCU}} = 0.0001\ \mu\mathrm{M\ s}^{-1}$.

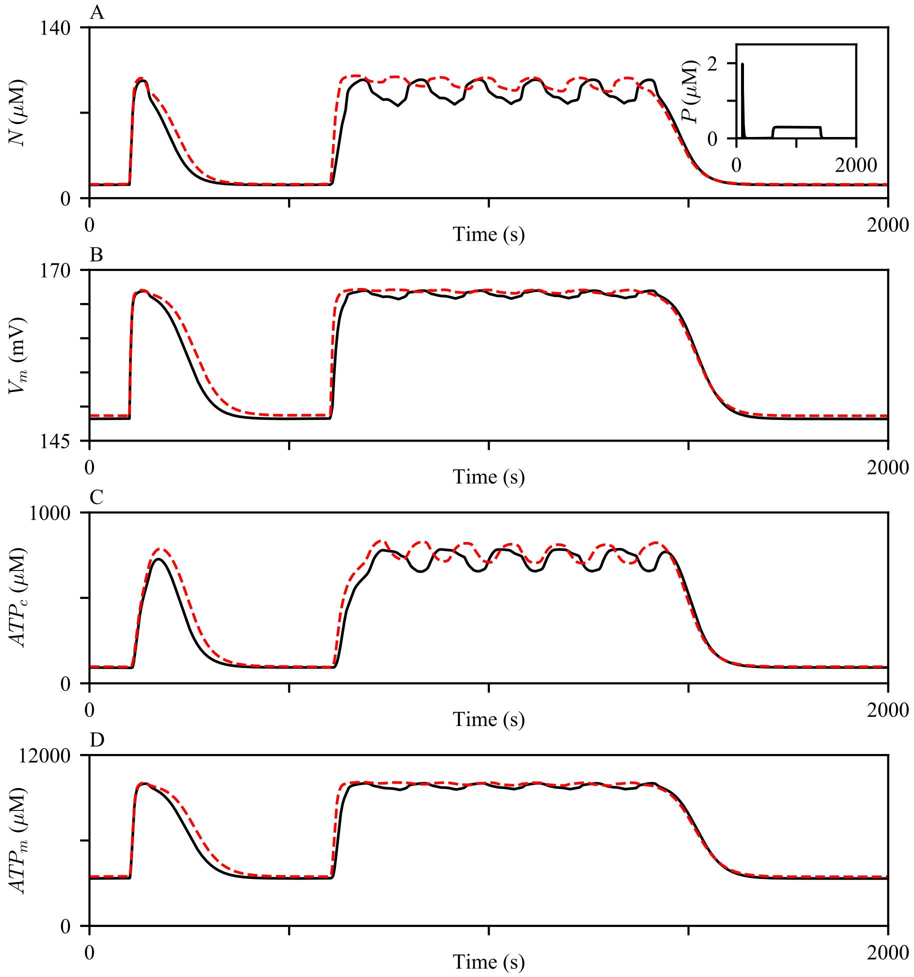

Figure 2: Mitochondrial metabolic variables, (A) N, (B) V_m, (C) ATP_c, and (D) ATP_m, generated by the control model (in black) and the obesity model (in dashed red). The models were given the same stimulation of IP_3, shown in the inset graph of (A).

References

1. Wacquier B, Combettes L, Tran Van Nhieu G, Dupont G. Interplay between intracellular Ca^{2+} oscillations and Ca^{2+}-stimulated mitochondrial metabolism. Sci Rep. 2016;6:19316. doi:10.1038/srep19316.
